# Structural and Functional Divergence of Growth Hormone-Releasing Hormone Receptors in Early Sarcopterygians: Lungfish and Xenopus

**DOI:** 10.1371/journal.pone.0053482

**Published:** 2013-01-04

**Authors:** Janice K. V. Tam, Billy K. C. Chow, Leo T. O. Lee

**Affiliations:** School of Biological Sciences, The University of Hong Kong, Pokfulam, Hong Kong; University of Rouen, France

## Abstract

The evolutionary trajectories of growth hormone-releasing hormone (GHRH) receptor remain enigmatic since the discovery of physiologically functional GHRH-GHRH receptor (GHRHR) in non-mammalian vertebrates in 2007. Interestingly, subsequent studies have described the identification of a GHRHR_2_ in chicken in addition to the GHRHR and the closely related paralogous receptor, PACAP-related peptide (PRP) receptor (PRPR). In this article, we provide information, for the first time, on the GHRHR in sarcopterygian fish and amphibians by the cloning and characterization of GHRHRs from lungfish (*P. dolloi* ***)*** and *X. laevis*. Sequence alignment and phylogenetic analyses demonstrated structural resemblance of lungfish GHRHR to their mammalian orthologs, while the *X. laevis* GHRHR showed the highest homology to GHRHR_2_ in zebrafish and chicken. Functionally, lungfish GHRHR displayed high affinity towards GHRH in triggering intracellular cAMP and calcium accumulation, while *X. laevis* GHRHR_2_ was able to react with both endogenous GHRH and PRP. Tissue distribution analyses showed that both lungfish GHRHR and *X. laevis* GHRHR_2_ had the highest expression in brain, and interestingly, *X. laevis*_GHRHR2_ also had high abundance in the reproductive organs. These findings, together with previous reports, suggest that early in the Sarcopterygii lineage, GHRHR and PRPR have already established diverged and specific affinities towards their cognate ligands. GHRHR_2_, which has only been found in xenopus, zebrafish and chicken hitherto, accommodates both GHRH and PRP.

## Introduction

Growth hormone-releasing hormone receptor (GHRHR) is a specific receptor for the growth hormone-releasing hormone (GHRH) in mammals which controls the synthesis and release of growth hormone (GH) from the anterior pituitary somatotrophs. Initially cloned from human, rat, and mouse pituitary, the isolated cDNA was found to encode a 423-amino acid protein with highly conserved sequence identities [1], [2], [3]. Later, orthologs from porcine [4], [5], bovine, and ovine [6] were also identified with several isoforms attributed to alternative RNA processing and carboxyl terminal truncation.

GHRHR belongs to the seven transmembrane secretin G-protein coupled receptor (GPCR) family, which also includes the receptors of secretin, glucagon, pituitary adenylate cyclase activating poplypeptide (PACAP), PACAP-related peptide (PRP), vasoactive intestinal peptide (VIP), parathyroid hormone (PTH) and calcitonin [7]. Previously, it was hypothesized that unlike mammals, PACAP was the sole physiological regulator of the growth axis in non-mammalian vertebrates (i.e. fish, amphibians and avians), since the GHRH-like peptides and their cognate receptors in these species were only weakly potent in GH stimulation [8]. In 2007, our group discovered a novel GHRH-GHRHR axis in teleosts (zebrafish and goldfish), showing that a “true” GHRH-GHRHR which is capable of stimulating GH secretion actually exists, while the GHRH-like ligand-receptor pair is in fact orthologs of vertebrate PRP-PRPR [9]. Meanwhile, an ortholog of this functional GHRHR was also cloned and characterized in chicken [10], [11]. Interestingly, in chicken *G. gallus*, a second GHRHR gene (GHRHR_2_) was found, although it only shared 46% amino acid sequence identity to the first GHRHR. A gene orthologous to the chicken GHRHR_2_ was also identified in *X. tropicalis* and zebrafish genomes, but not in mouse, rat and human [12].

Reports on the functional characterization of GHRHRs hitherto have been limited to teleosts and avians in the non-mammalian vertebrate lineage. To understand the structural and functional shaping of GHRHR prior the divergence of tetrapods, we have chosen lungfish *Protopterus dolloi*, one of the few extant finned sarcopterygians which is sister group to the living tetrapods that emerged in the Devonian after the sarcopterygian-actinopterygian split [13]. Whereas little is known of the existence of GHRHR or its cognate ligand in lungfish, our group has proved the presence of a GHRH peptide in *Xenopus laevis* [9]. However, we were not able to find a functional GHRHR at that time. The presence of a functional GHRHR which is orthologous to the mammalian GHRHRs remains equivocal: A partial GHRHR-like sequence in the *X. tropicalis* genome has been reported [12], but its true identity has never been characterized. In the present work, revealed by characterizing GHRHR structurally and functionally in lungfish and *Xenopus laevis*, and taking together the information of other GHRHR-related receptors in literature, we showed that GHRHR in lungfish was functionally similar to mammalian and teleost GHRHR as suggested by its high affinity towards GHRH, whereas GHRHR_2_ in *Xenopus* is acting as the cognate receptor for both GHRH and PRP peptides.

## Results

### Identification and Analyses of Putative GHRH Receptors in *P. dolloi* and *X. laevis*

Primers were designed according to the predicted partial sequence from *X. tropicalis* genome for the amplification of the putative GHRHR in *X. laevis*. A full-length *X. laevis* GHRHR (xGHRHR_2_) cDNA (Genbank accession no. JN378527) of 1835 bp with an open reading frame of 1269 bp encoding a 423-amino acid protein with a 29-amino acid signal peptide was obtained (Figure S1). Based on all the GHRHR sequences available, degenerative primers were designed for the amplification of GHRHR in *P. dolloi*. The full-length putative *P. dolloi* GHRHR (lfGHRHR) cDNA (Genbank accession no. JN378526) was 2191 bp with an open reading frame of 1308 bp encoding a 436-amino acid protein with a 29-amino acid signal peptide (Figure S2). All the primer sequences were shown in Table S1.

Unlike PACAP/VIP receptors, vertebrate GHRHRs only displayed moderate sequence identity conservation. When the putative amino acid sequence of lfGHRHR was compared to the human, mouse, chicken and goldfish GHRHR sequences (Figure S3), they shared 45.9–55.1% identity with the highest in chicken GHRHR (55.1%). More importantly, most of the structural features of GHRHRs, including the 7 cysteine residues and the glycosylation site in the N-terminus, are conserved. When the putative amino acid sequence of xGHRHR_2_ was compared to the teleost fish and chicken GHRHR_2_, GHRHR and PRPR sequences (Figure S4), xGHRHR_2_ shared the highest identity with zebrafish and chicken GHRHR_2_ sequences (52.1% and 64.8% respectively), lower sequence identity with the PRPR sequences (40.9% to 47.6%), and the lowest with GHRHR sequences (40.3% to 40.7%).

**Figure 1:**
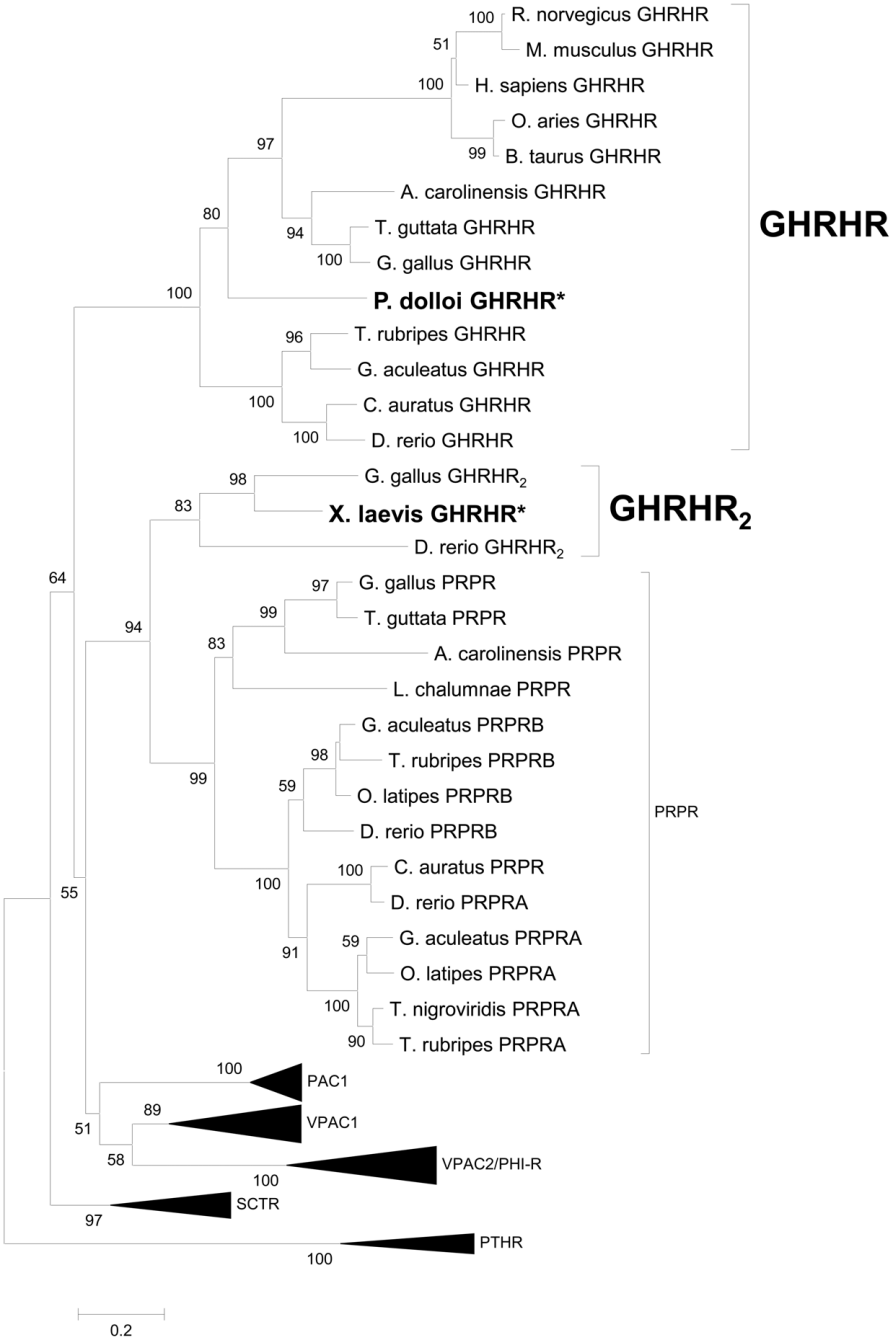
Evolutionary analysis of the osteichthyans secretin GPCR family. The maximum likelihood (ML) optimal tree topology is presented and was constructed with MEGA5. ML bootstrap values higher than 50% are indicated at nodes. To facilitate interpretation, PTHR was used as an outgroup based on the proposed models for secretin GPCR family evolution [7]. The tree supported the identities of lfGHRHR and xGHRHR_2_ as the orthologs of mammalian GHRHR and chicken GHRHR_2_ respectively. Accession numbers of the sequences used were listed in Table S2.

To verify the identities of lfGHRHR and xGHRHR_2_ in the secretin GPCR family, we performed phylogenetic analyses using the Maximum Likelihood method with the Jones-Taylor-Thornton (JTT) model (Figure 1). In agreement with the sequence alignment, lfGHRHR was grouped to the GHRHR clade, with a topology consistent with the established phylogeny of the Osteichthyes lineage [14], while xGHRHR_2_ was grouped to a separate clade that included both PRPR and GHRHR_2_ sequences, and formed a sub-clade with zebrafish and chicken GHRHR_2_s. The overall topology of the tree was consistent with the previous reports [7], [15], that GHRHR and PRPR are the closest paralogous genes in the secretin GPCR family. Taken together, these data supported the identity of lfGHRHR as an ortholog of the other vertebrate GHRHRs, whereas the xGHRHR_2_ is an orthologous receptor of the zebrafish and chicken GHRHR_2_.

### Functional Analyses of Putative *P. dolloi* and *X. laevis* GHRH Receptors

**Figure 2:**
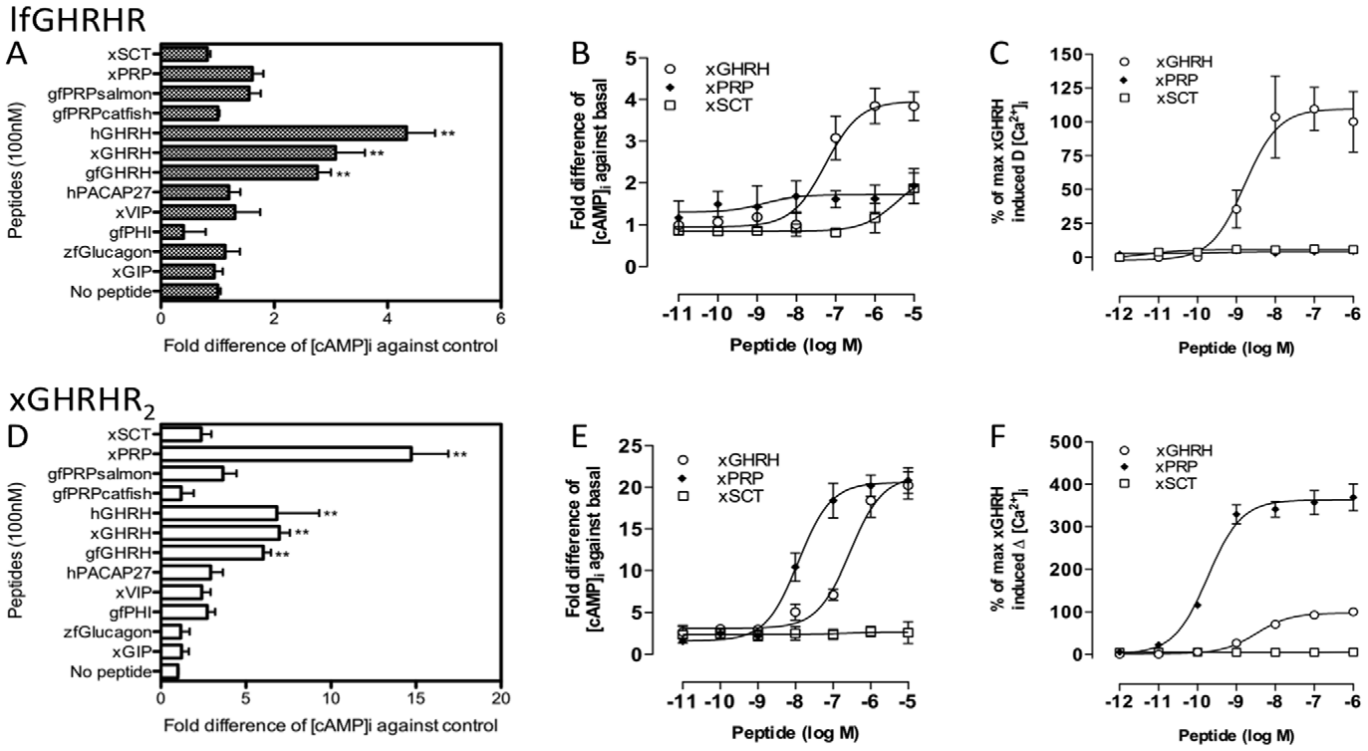
Functional characterization of lfGHRHR and xGHRHR_2_. Intracellular cAMP accumulation ([cAMP]_i_) in response to 100 nM of GHRH and related peptides on CHO-K1 cells transfected with (A) lfGHRHR and (D) xGHRHR_2_ (*** indicates *P*<0.001, ** indicates *P*<0.01, and * indicates *P*<0.05). Effects of GHRH and related peptides on graded concentrations of peptides on [cAMP]_i_ (B) lfGHRHR- and (E) xGHRHR_2_-expressing cells. The intracellular calcium mobilization ([Ca^2+^]_i_) assays of (C) lfGHRHR- and (F) xGHRHR_2_-expressing cells. For [cAMP]_i_, values represent mean ± SEM (n = 4). For ([Ca^2+^]_i_, data were expressed in ΔRFU value (maximum changes in the fluorescence signals from baseline) and converted to percentage of the maximum of xGHRH-induced [Ca^2+^]_i_ elevation. Results are expressed as mean ± SEM from at least 10 independent experiments, cell number = 20 to 50. Peptide species: h, human; x, *X. laevis*, zf, zebrafish *D. rerio*; gf, goldfish *C. auratus*.

When lfGHRHR-CHO cells were stimulated with GHRH and related peptides at 100 nM, all the tested GHRHs (human, xenopus and goldfish) were able to stimulate significant increase in [cAMP]i (2.8 to 4.2 fold increase of control) (Figure 2A). No activation was observed in other peptide treatments, including PRPs. As no endogenous lungfish ligands have been reported and the xGHRH has demonstrated significant activation in the peptide screening assay, xenopus GHRH, PRP and SCT were chosen for the subsequent analyses. Graded concentrations of xGHRH stimulated lfGHRHR in a dose-dependent manner (Figure 2B) with EC_50_ = 56.1 nM. Similar to the peptide screening results, xSCT and xPRP were not able to stimulate lfGHRHR. In the calcium mobilization assay, consistent with the cAMP assay, graded concentrations of xGHRH stimulated intracellular calcium accumulation ([Ca^2+^]_i_) dose-dependently (Figure 2C) with EC_50_ = 1.66 nM. Also shown by the real-time traces of single cells (Figure S5), xGHRH evoked the strongest peak of calcium elevation, while xSCT and xPRP were not able to activate the lfGHRHR. Taken together, our data demonstrated that GHRH was the most potent ligand in stimulating lfGHRHR.

The recombinant xGHRHR_2_ was tested with its potentially endogenous ligands, xGHRH and xPRP, and related peptides at 100 nM. As shown by Figure 2D, apart from xGHRH (7.0 fold increase of control) and xPRP (14.8 fold increase of control), hPACAP27, xVIP, gfPHI and gfPRPsalmon could also stimulate [cAMP]_i_ in xGHRHR_2_, though with weak potencies (from 2.4 to 3.7 fold increase of control). Graded concentrations (10^−11^ to 10^−5 ^M) of xGHRH and xPRP stimulated xGHRHR_2_ dose-dependently while xSCT peptide was unable to stimulate xGHRHR_2_ (Figure 2E). Consistently, calcium signaling of xGHRHR_2_ was activated by nanomolar level of xPRP (EC_50_ = 0.19 nM) and xGHRH (EC_50_ = 3.28 nM) (Figure 2F and Figure S5). These results indicated that xGHRHR_2_ could be activated by the endogenous PRP as well as GHRH by utilizing both cAMP and calcium pathways.

### Tissue Expression of *P. dolloi* and *X. laevis* GHRH Receptors

**Figure 3:**
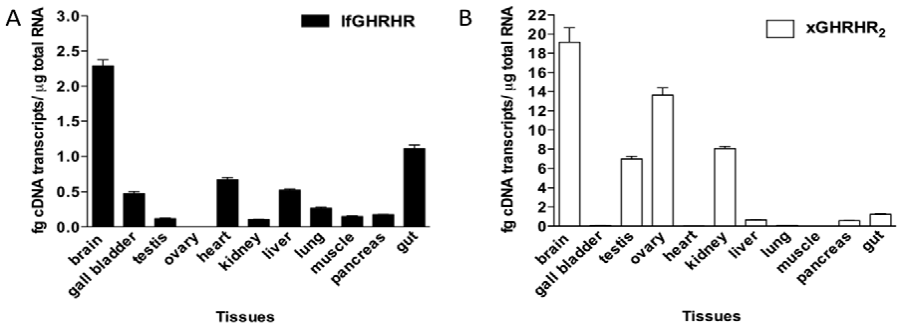
Tissue distribution profile of lfGHRHR and xGHRHR2. Gene expression of (A) lfGHRHR in *P. dolloi* and (B) xGHRHR_2_ in *X. laevis* was assessed by quantitative real-time PCR. The expression level of each gene was calculated from respective standard curve. Data are expressed as mean ± SEM (n = 4). Highest expression for both GHRH receptors was detected in the brain, as observed in mammals.

Tissue distribution of GHRH receptors in *P. dolloi* and *X. laevis* were compared by quantitative real-time PCR. Similar to the distribution pattern of mammalian GHRHRs [3], [16], [17], highest expression for both transcripts was detected in brain, and relatively lower expression in peripheral tissues, except *X. laevis* muscle (Figure 3A and 3B). Interestingly, our data showed that xGHRHR_2_ was highly expressed in the reproductive organs of *X. laevis* but not in lfGHRHR. This differential distribution pattern suggested that the amphibian GHRHR may have physiological importance in the reproductive system. Since we have demonstrated that xGHRHR_2_ was also a specific receptor for xPRP, together with our previous report on the reproductive bioactivity of PRP in goldfish [18], the high abundance of GHRHR_2_ in the gonads suggested that xGHRHR_2_ could be involved in the *X. laevis* reproductive axis, in addition to modulating growth in the frog.

### In Silico Genomic Locations of GHRHR

**Figure 4:**
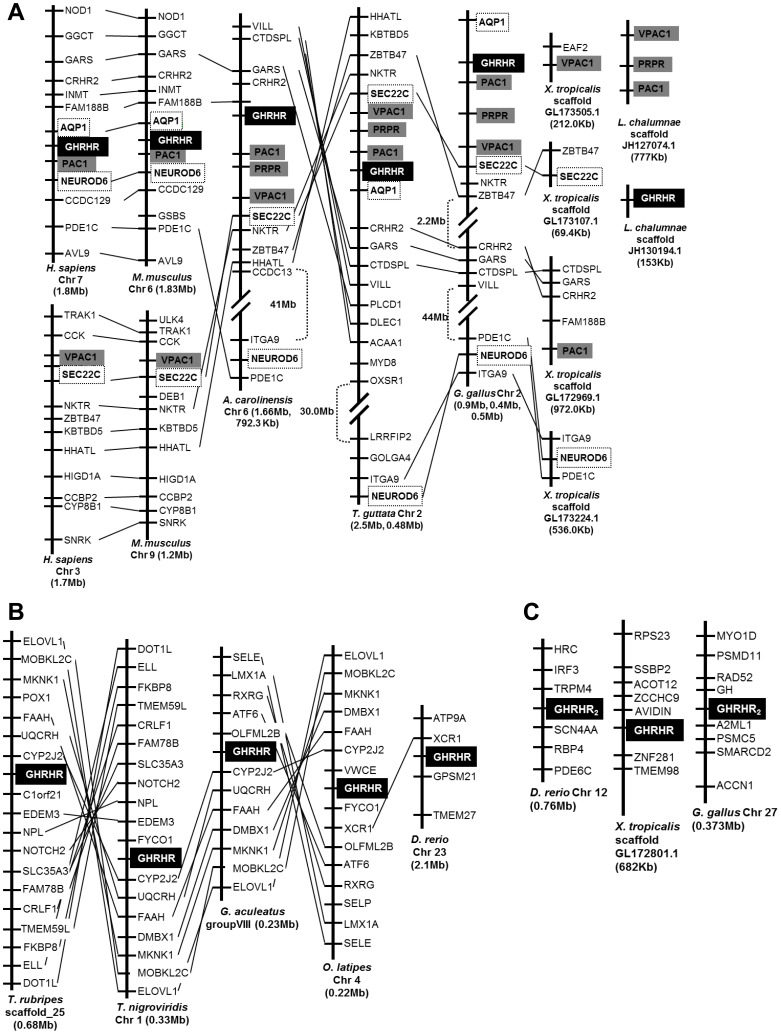
Gene linkage comparisons of GHRHRs in the Osteichthyes lineage. Genes in vicinity of GHRHR were mapped, and syntenic genes were linked by straight lines. Size of the chromosomal region analyzed was given underneath based on the current edition of Ensembl databases. Syntenic genes encoding other secretin GPCR receptors were drawn in grey boxes, and the conserved flanking genes of GHRHR were drawn in closed boxes. (A) Gene environment of GHRHR in the Sarcopterygii lineage represented by human, mouse, lizard, chicken, frog and coelacanth was compared. Despite the syntenic genomic locations of GHRHR and neighbouring genes from human to avians, GHRHR was not located in frog. (B) Gene environment of GHRHR in the Actinopterygii lineage represented by fugu, tetraodon, stickleback, medaka, and zebrafish. Apart from the less conserved genomic region of zebrafish GHRHR, genes in proximity of other teleost GHRHRs were highly syntenic. However, they displayed an entirely different gene environment when compared to the sarcopterygian GHRHRs. (C) Genomic location analysis of xGHRHR characterized in present work and GHRHR_2_ in zebrafish and chicken. Gene synteny could neither be identified inter-species nor between the two GHRHR genes in the same species. The figures were not drawn to scale.

To further investigate the evolutionary divergence of GHRHR, genomic locations of GHRHRs were compared for their chromosomal syntenies in osteichthyans. GHRHRs in mammals (human and mouse), non-mammalian tetrapods (lizard, avians and frog), coelacanth and teleosts (fugu, tetraodon, stickleback, medaka and zebrafish) in the Actinopterygii lineage were mapped (Figure 4).

Comparison of the gene environment of GHRHR genes in avians, lizard, and mammals along the Sarcopterygii lineage revealed that, GHRHR genes are always located next to PAC1 genes (Figure 4A). Apart from PAC1 genes, AQP1 and NEUROD6 are located in the vicinity of GHRHR genes in mammals (Figure 4A). In avians and lizard, NEUROD6 becomes more distantly located from GHRHR despite in the same chromosome. Interestingly, in *X. tropicalis*, although the orthologues of the GHRHR flanking genes, such as CTDSPL, GARS, and CRHR2 on scaffold GL172969.1, and ITGA9, NEUROD6, and PDE1C on scaffold GL173224.1, were conserved in the short-range gene order based on the present assembly of the frog genome (*X. tropicalis* JGI4.2), an orthologous GHRHR gene could not be located in the proximity of these scaffolds.

Since the genome of lungfish is not available at present, we have analyzed another early diverging sarcopterygian – coelacanth *L. chalumnae* [13]. Based on the present assembly of the coelacanth genome (LatCha1), VPAC1 (ENSLACP00000004890), GHRHR (ENSLACP00000014088) and VPAC2 (ENSLACP00000015179) were predicted to be present on scaffold JH127074.1 by Ensembl (Figure 4A and Table S2). However, sequence alignment and phylogenetic analyses (data not shown) revealed that the predicted coelacanth GHRHR (ENSLACP00000014088) is in fact orthologous to the characterized PRPRs in non-mammalian vertebrates (Figure 1), whereas the predicted coelacanth VPAC2 (ENSLACP00000015179) is orthologous to mammalian PAC1. To check for the presence of GHRHR in coelacanth, we performed a BLAST search using the lfGHRHR. Short fragments displaying high sequence identity (93 to 100%) to the lungfish GHRHR were found on scaffold JH130194.1. However, due to the incompleteness of the sequence fragments, we were not able to assemble these fragments to a full-length GHRHR sequence that returned highly reliable phylogenetic analyses.

In teleosts, represented by fugu *T. rubripes*, tetraodon *T. nigroviridis*, stickleback *G. aculeatus*, medaka *O. latipes*, and zebrafish *D. rerio*, the gene environment of GHRHR was entirely different from the mammalians as well as the non-mammalian sarcopterygians (Figure 4B). Except zebrafish, the neighbouring genes of GHRHR were highly conserved among the teleosts. In zebrafish, only one gene, XCR1, was conserved. In fugu and stickleback, XCR1 gene was found to be adjacent to PAC1/PRPR instead (Figure S6). The distinctly less syntenic zebrafish GHRHR gene could be attributed to the divergence of zebrafish, which belongs to the Ostariophysi lineage, basal to the Acanthomorpha species to which fugu, tetraodon, stickleback, and medaka belong [19], [20].

In 2009, a thorough analysis on the GPCR family in the *X. tropicalis* genome has been reported [15]. Using the JGI *Xenopus tropicalis v4.1* database, the authors deciphered the identities of the majority (about 95%) of GPCR repertoire in *X. tropicalis*. The protein sequences predicted from these genes were phylogenetically analyzed with the related receptors in human; one of which, named GHRHR 342326 (partial sequence, 290 amino acid residues), was identified as the homologue of human GHRHR.

Aside from this, a second GHRHR gene (GHRHR_2_) has been reported to exist in the genomes of zebrafish, *X. tropicalis* (partial sequence, 291 amino acid residues), and chicken, but not in mammals, among which the chicken GHRHR_2_ was functionally characterized [12]. Both chicken GHRHRs were shown to be specific receptors of its endogenous ligand, chicken GHRH, and exhibited highly similar ligand affinity profiles, despite their predicted amino acid sequences only shared 46% identity [12].

Based on these information, the putative xGHRHR_2_ cloned in this study was aligned with the *X. tropicalis* GHRHR 342326 and *X. tropicalis* GHRHR_2_ using their predicted amino acid sequences (data not shown). It was found that both reports were actually referring to the same protein encoded from the same gene, and their sequences were showing 97.0% identity to the putative xGHRHR_2_ cloned in the present work. To verify this, a BLAST search was performed to locate the orthologous gene of xGHRHR_2_ in the *X. tropicalis* genome. In depth analysis of the best matches revealed that the corresponding gene in *X. tropicalis* was the partial GHRHR_2_ reported previously [12], [15]. Substantiated by these analyses, it can be concluded that the putative xGHRHR_2_ identified in this report is equivalent to the *X. tropicalis* GHRHR 342326 [15] and xGHRHR_2_ [12].

To understand the true identity of xGHRHR, the gene environment of GHRHR (Figure 4A and 4B) and GHRHR_2_ in zebrafish, *X. tropicalis* and chicken were compared (Figure 4C). However, gene synteny could neither be identified inter-species nor between the two GHRHR genes in the same species. When the genome regions of chicken and zebrafish GHRHR_2_ were compared to their corresponding PRPR genes, no gene linkages could be identified (Figure 4C and S6). To further explore the divergence of GHRHR_2_, we have searched for the presence of GHRHR_2_ in all the available osteichthyan genomes. However, none of the osteichthyan genomes contained any sequence that highly resembled the zebrafish, xenopus and chicken GHRHR_2_. Hence, according to the present version of all the osteichthyan genomes, GHRHR_2_ only exists in zebrafish, xenopus and chicken.

## Discussion

### Does Lungfish GHRHR have an Endogenous Ligand?

Substantiated by the sequence alignment and phylogenetic analyses, lfGHRHR is showing a structural similarity to the mammalian GHRHRs. Functional characterization of lfGHRHR also revealed that, like its mammalian counterparts, lfGHRHR has a higher affinity to GHRH than other peptides in the secretin/glucagon superfamily. From the recently released coelacanth genome, we have also identified a GHRH sequence predicted by the present genome assembly (Figure S7). Alignment of the coelacanth predicted mature GHRH peptide with the other non-mammalian GHRHs revealed high conservation of sequence identity, amongst which teleost GHRHs were 96.3% identical to coelacanth GHRH with one amino acid (residue 26) difference only. Based on the close phylogenetic relationship of coelacanth and lungfish, both of which are early diverging extant sarcopterygians [13], [21], [22], and the high affinity of lfGHRHR to all the GHRH peptides tested, we hypothesized that, a GHRH sequence that structurally resembles fish GHRH exists in lungfish.

### Diverged Functions of GHRHR, GHRHR_2_ and PRPR

**Figure 5:**
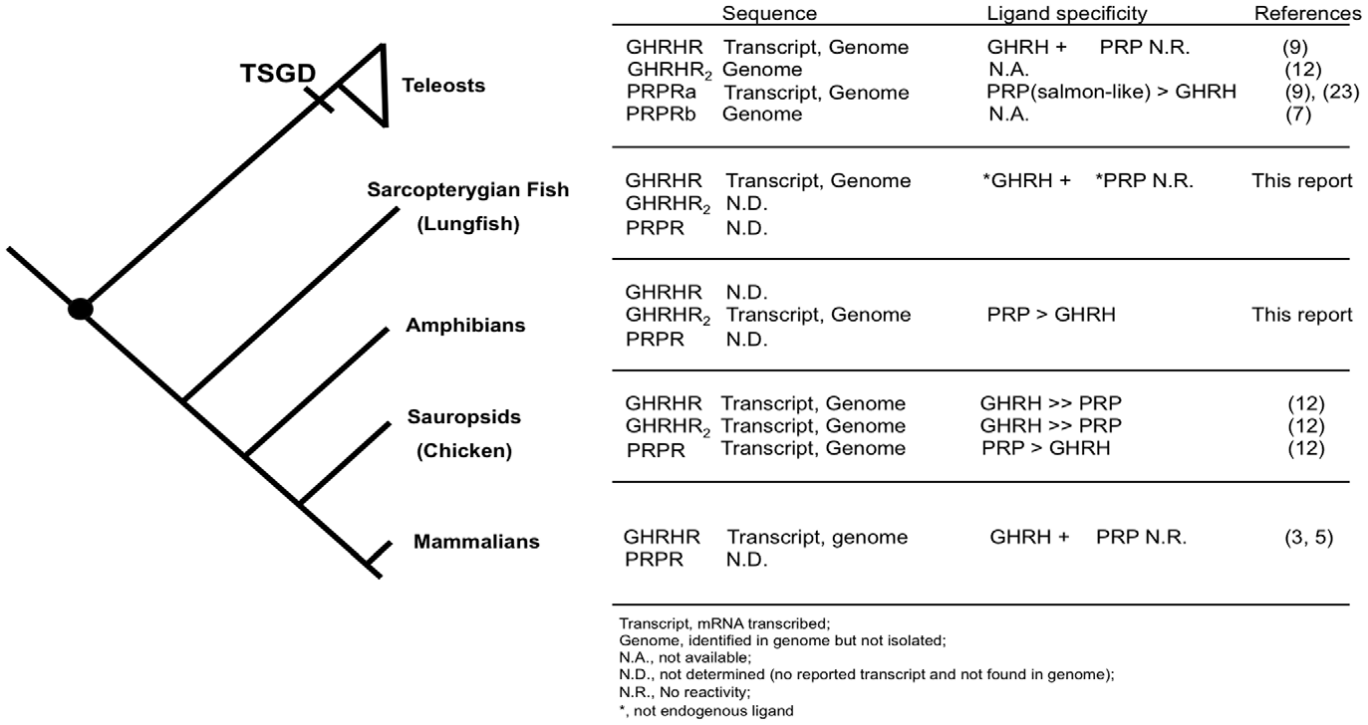
GHRHR, GHRHR2 and PRPR in Osteichthyes lineage. Identified GHRHR, GHRHR2, PRPR and their ligand specificity were summarized in each taxon group. The Sarcopterygii-Actinopterygii split was marked by a black dot (•) and the timing of TSGD (Teleost-specific genome duplication) was indicated [14]. Abbreviations and terminology: Transcript refers to mRNA transcribed; Genome means identified in genome but not isolated; N.A., not available; N.D., not determined (no reported transcript and not found in genome); N.R., No reactivity; *, not endogenous ligand.

Figure 5 summarized all the identified GHRHR, GHRHR_2_, and PRPR in each taxon group of the Osteichthyes lineage. GHRHR gene is present in teleosts, birds, and mammals; whereas another GHRHR-like, GHRHR_2_ gene is also present in the Teleostei and avian (chicken). In this study, for the first time, the sequences of GHRHR in lungfish and GHRHR_2_ in *X. laevis* (amphibian) have been identified. For PRPR, there are two isoforms (a and b form) in teleosts [23], one of which was a duplicate diverged during the TSGD. Aside from teleosts, PRPR has only been identified and characterized in avian at present.

When the ligand affinities of all the characterized osteichthyan GHRHRs were compared, GHRHR demonstrated high specificity towards its cognate ligand, GHRH, in both Sarcopterygii and Actinopterygii lineages. Although in chicken, GHRHR showed a slight activity for PRP, it was approximately 245 fold less potent than the endogenous GHRH in stimulating GHRHR [12], which was similar to the potencies of other secretin/glucagon superfamily peptides due to cross-reactivity [3], [6]. Suggested by these findings, the ligand affinities of GHRHR to GHRH could have been well-defined and established prior the Sarcopterygii-Actinopterygii split.

In the secretin GPCR family, since GHRHR and PRPR are phylogenetically the closest sister genes [7], PRP (GHRH-like) was regarded as the only physiological GHRH in non-mammalian vertebrates before the discovery of authentic GHRH by our group in 2007 [9]. Functionally, PRP only displayed weak and inconsistent GH-releasing activities in teleosts and avians [8]. Recent reports provided evidence that, instead of controlling GH secretion, PRP-PRPR regulates reproductive functions via the brain-pituitary-gonadal (BPG) axis [18], [19]. In teleost fish, stimulation of PRPR by its endogenous PRP regulated the gene expression of salmon gonadotropin-releasing hormone (sGnRH), follicle-stimulating hormone (FSH) and luteinizing hormone (LH) in the BPG axis, whereas GHRH controls growth hormone release via GHRHR in teleost [9] as well as mammals [1]–[6].

In addition to the GHRHRs and PRPRs, another closely related receptor GHRHR_2_ was also identified in teleost, amphibian and avian. However, different from avian and teleosts, GHRHR and PRPR co-exist with GHRHR_2_, while results from the present study showed that in *X. laevis* and *X. tropicalis*, only one gene, the GHRHR-like or the GHRHR_2_, is structurally and functionally a potential candidate that is orthologous to the mammalian GHRHR genes. Phylogenetically, the presently cloned xGHRHR is most closely related to the chicken and zebrafish GHRHR_2_ genes rather than the GHRHRs or the PRPRs (Figure 1). It has been proposed that these GHRHR_2_ genes have already emerged before the fish-tetrapod split because they are present in the representative species of the teleosts, amphibians, and avian [12]. However, the lack of chromosomal synteny among these genes and absence of orthologs in other osteichthyan species challenged this hypothesis. Instead of descending from a common ancestor, these genes could have diverged in the lineage-specific events, hence are only found in several species. In the case of xGHRHR_2_, since *Xenopus* species give rise to new species by allopolyploidization, which is a type of genome duplication that can result from hybridization among species [24], genes could be easily introduced or deleted during these events. Suggested by the data in this study that the xGHRHR_2_ is able to react with both endogenous GHRH and PRP peptides, it may function as a GHRH and PRP receptor simultaneously and hence has a structural resemblance of a GHRHR as well as a PRPR. The potential dual function of xGHRHR_2_ is also suggested by its major sites of expression: its high abundance in brain as all GHRHRs and in reproductive organs as all PRPRs. Therefore, it is highly possible that GHRH and PRP are utilizing the same receptor, xGHRHR_2_ in *X. laevis*. This hypothesis is further supported by the absence of GHRHR and PRPR genes in the *in silico* genomic locations analysis (Figure 4). Nevertheless, we could not exclude the possibility that in other amphibians, extra copies of GHRHR or PRPR exist.

## Materials and Methods

### Ethics Statement

I confirm that all the animal treatments conducted in this study has been approved by the review board of the University of Hong Kong, the Committee on the Use of Live Animals in Teaching and Research (CULATR), with an approval ID of 1496-07.

We also clarified the ethics committee (“Committee on the Use of Live Animals in Teaching and Research” from University if Hong Kong) that approved our animal research and government approval (Department of Health) in the ethics statement:

All animal treatments were in accordance with the guidelines established by the Committee on the Use of Live Animals in Teaching and Research (CULATR, Approval ID 1496-07) of the University of Hong Kong with the Cap. 340 animal license issued by the Department of Health of the Hong Kong Government under the Animals Ordinance.”

Committee on the Use of Live Animals in Teaching and Research (CULATR) is the official committee in University of Hong Kong that monitor all the teaching and research experiments involving living vertebrate animals. Our work also approved by Hong Kong government (the Department of Health) under the Animals Ordinance Cap 340.

### Animals and Peptides

Lungfish *P. dolloi* (32–40 cm) was purchased from a local commercial supplier, and *X. laevis* (Male: 8 to 11 cm; female: 9 to 14 cm) was bought from Xenopus I (Xenopus I, Inc., CA)**.** All the animals used in this study were at adult stage and sexually matured. Glucagon, glucagon-like peptides, GIP, GHRH, PRP, PACAP peptides were ordered from the Proteomics Resource Center of the Rockefeller University (http://proteomics.rockefeller.edu/). VIP and PHI peptides were synthesized by Bachem California (Bachem California, Inc., CA). Human SCT was bought from AnaSpec (AnaSpec, Inc., CA), and xSCT was a gift from Prof. Hubert Vaudry (University of Rouen, France). All synthetic peptides were of >95.0% purity.

### Total RNA Extraction and First-strand cDNA Synthesis

Animals were sacrificed by cervical decapitation. Total RNA was isolated from freshly excised tissues by TriPure reagent (Invitrogen, Carlsbad, CA) as described in previous report [25]. First-strand cDNA from 5 µg total RNA was synthesized according to the protocol of SuperScript III Reverse Transcriptase (Invitrogen) using the Adaptor Primer (AP) (Invitrogen).

### Molecular Cloning of GHRH Receptors from *P. dolloi* and *X. laevis*

Primers for the amplification of *X. laevis* GHRHR-like sequence were designed based on the partial sequences obtained from the *X. tropicalis* genome by a BLAST search. Degenerate primers for the amplification of lungfish GHRHR (lfGHRHR) were designed according to conserved regions of aligned GHRHR sequences (Table S1). Rapid amplification of cDNA ends (RACE) was performed using the 5′ and 3′ RACE amplification kits (Invitrogen) with specific primers designed according to the partial sequences. Full-length cDNA clones encompassing the 5′ to 3′ untranslated regions were produced by PCR with specific primers and confirmed by DNA sequencing. Full-length GHRHR cDNAs were subcloned to pcDNA3.1 (+) (Invitrogen) for functional expression. All sequences newly identified in the present study have been deposited in the GenBank (Genbank accession no.: lfGHRHR, JN378526; xGHRHR_2_, JN378527).

### Tissue Distribution of GHRHR in *P. dolloi* and *X. laevis*

Quantitative real-time PCR was used to determine the expression profiles of GHRH receptors in *P. dolloi* and *X. laevis* in various tissues. First-strand cDNAs were synthesized from total RNA as previously mentioned (Materials and Methods: Total RNA Extraction and First-strand cDNA Synthesis). RT-PCR (n = 4, each in duplicates) was performed using the Power SYBR Green PCR Master Mix (Applied Biosystems, Foster City, CA) and the 7300 Real Time PCR System (Applied Biosystems). Primers used in the real-time PCR are listed in Table S1. The threshold cycle (Ct) is defined as the fractional cycle number at which the fluorescence reaches 10-fold standard deviation of the baseline (from cycle 3 to 10). The specificity of the SYBR PCR signal was confirmed by both melt curve analysis and agarose gel electrophoresis. Standard curves were established by 10x serial dilution of respective plasmid stocks [25].

### Transient Expression of GHRH Receptors in CHO cells

Chinese Hamster Ovary (CHO) cells (ATCC, Manassas, VA) were cultured in MEM/10% FBS/100 U/ml Penicillin/100 g/ml Streptomycin on 100 mm tissue culture plates at 37°C and 5% CO_2_ until 80% confluence. GHRHR expression construct (2 µg) was used to transfect 1×10^5^ CHO cells with 6 µl GeneJuice reagent (Novagen, Darmstadt, Germany). A control cell line was established by transfecting the cells with the pcDNA 3.1 (+) vector (Invitrogen). Intracellular cAMP production upon peptide stimulation was measured using the LANCE cAMP assay kit (Perkin-Elmer, Waltham, MA) in the Victor X4 multilabel reader (Perkin-Elmer) according to the manufacturer’s protocol. Intracellular cAMP levels ([cAMP]_i_) were measured and expressed as cAMP concentration relative to the basal level (stimulation buffer alone without peptide addition). Negative control experiments were performed by simultaneous peptide stimulation at 10 µM on the control cell line in each experimental trial.

For confocal calcium imaging, transiently transfected cells were plated at a density of 3000 cells/well in 24-well plates (Sigma-Aldrich, St. Louis, MO). After overnight incubation, cells were pre-loaded with 5 µM Fluo-3 acetoxymethyl ester (AM) (Sigma) for 45 min at 37°C in Tyrode solution consisting of (mM): 140 NaCl, 5 KCl, 1 MgCl_2_, 1 CaCl_2_, 10 glucose and 10 HEPES at pH 7.4. Calcium transient of single receptor-transfected CHO cell was recorded with a confocal imaging system (Olympus Fluoview System version 4.2 FV300 TIEMPO) mounted on an upright Olympus microscope (IX71). Peptides at concentrations ranging from 10^−5^ to 10^−12^ M were added at designated time point and calcium level was traced in a real-time manner using the Fluoview software (Olympus). Data were expressed in ΔRFU value (maximum changes in the fluorescence signals from baseline) and converted to percentage of the maximum of xGHRH-induced [Ca^2+^]_i_ elevation (i.e. xGHRH [Ca^2+^]_i_ at 10 µM = 100%). For both assays, ionomycin (10 µM) was added at the end of each experiment to test the vitality of the cells.

### Phylogenetic Analysis

Amino acid sequences were aligned with Clustal X. Phylogenetic trees and their best-fit models were generated by MEGA 5.0 [26]. The trees were calculated by Maximum Likelihood method with the Jones-Taylor-Thornton (JTT) model and combined with +G: rate heterogeneity among sites. 1000 bootstrap replicates were used to assess nodal support. Sequences used in these analyses and their accession numbers retrieved from Genbank and Ensembl were listed in Table S2.

### Chromosomal Synteny Analysis

Gene environment of osteichthyan GHRHRs was mapped and compared to identify conserved gene blocks. Genomes were searched on the Ensembl using the current databases available, including human GRCh37, mouse NCBIM37, Anole Lizard (*Anolis carolinensis*) AnoCar2.0, Zebra Finch (*Taeniopygia guttata*) taeGut3.2.4, chicken WASHUC2.1, *X. tropicalis* JGI4.2, coelacanth (*Latimeria chalumnae*) LatCha1, Fugu (*T. Rubripes*) FUGU4, Tetraodon (*T. Nigroviridis*) TETRAODON8, Stickleback (*G. aculeatus*) BROADS1, medaka (*O. latipes*) MEDAKA1, and zebrafish (*D. rerio*) Zv9.

### Statistical Analysis

Results are presented as mean ± SEM, and are averages of the means of duplicated assays in at least three independent experiments. GraphPad Prism version 5.0 (GraphPad Software, Inc., San Diego, CA) was used to plot the sigmoidal curves in the cAMP and calcium mobilization assays and to perform statistical analyses using one-way ANOVA followed by Dunnett’s test. Differences were considered significant when *P*<0.05.

## Supporting Information

Figure S1 ***X. laevis*** **growth hormone-releasing hormone receptor 2 (xGHRHR_2_) nucleotide (GenBank accession no. JN378527) and deduced amino acid sequences.** Nucleotides (lower line) and amino acids (upper line) were numbered from the initiation methionine. The full-length cDNA was 1835 bp with an open reading frame of 1269 bp encoding a 423-amino acid protein. The signal peptide (29 amino acids) was indicated in bold characters. Transmembrane domains were underlined with solid lines. These structural features were determined by software from the CBS Prediction Servers (http://www.cbs.dtu.dk/services/).
(PPTX)

Figure S2 **Lungfish** ***P. dolloi*** **growth hormone-releasing hormone receptor (lfGHRHR) nucleotide (GenBank accession no. JN378526) and deduced amino acid sequences.** Nucleotides (lower line) and amino acids (upper line) were numbered from the initiation methionine. The full-length cDNA was 2191 bp with an open reading frame of 1308 bp encoding a 436-amino acid protein. The signal peptide (29 amino acids) was indicated in bold characters. Transmembrane domains were underlined with solid lines.
(PPTX)

Figure S3 **Growth hormone-releasing hormone receptor (GHRHR) protein sequence alignment with lfGHRHR.** Identical and conserved amino acid residues were written and highlighted in orange and blue respectively. Putative transmembrane domains were overlined and labeled. # and * indicate potential sites for N-linked glycosylation and conserved cysteine residues, respectively. Gaps (represented by - ) were introduced to maximize sequence homology. Percent amino acid identity and homology were listed in respect to lungfish GHRHR.
(PPTX)

Figure S4 **Amino acid sequence comparison of** ***X. laevis*** **growth hormone-releasing hormone receptor (xGHRHR) to zebrafish** ***D. rerio*** **and chicken** ***G. gallus*** **GHRHR_2_ and PRPR.** Identical and conserved amino acid residues were written and highlighted in orange and blue respectively. Putative transmembrane domains were overlined and labeled. # and * indicate potential sites for N-linked glycosylation and conserved cysteine residues, respectively. Gaps (represented by - ) were introduced to maximize sequence homology. Percent amino acid identity and homology were listed in respect to *X. laevis* GHRHR.
(PPTX)

Figure S5 **Representative traces of xSCT, xGHRH and xPRP on intracellular calcium mobilization in lfGHRHR, xGHRHR and null pcDNA 3.1-transfected CHO cells.** Peak magnitude of traces was proportional to the order of potency of the ligands tested. Traces were obtained from at least 10 calcium assays with respective control shown on the right panel. Ionomycin (10 µM) was added at the end of each experiment to test the vitality of the cells.
(PPTX)

Figure S6 **Chromosomal synteny analysis of GHRHR and PRPR in osteichthyans.** Genes in vicinity of GHRHR and/or PRPR were mapped, and syntenic genes were linked by straight lines. Size of the chromosomal region analyzed was given underneath based on the current edition of Ensembl databases. Syntenic genes encoding secretin GPCR receptors were drawn in bold, NEUROD6 and XCR1 were written in colour fonts for easy referencing to main text.
(PPTX)

Figure S7 **Amino acid sequence comparison of GHRH. The coelacanth GHRH was predicted from the newly released genome sequence.**
(PPTX)

Table S1 **List of primers used in PCR and real-time PCR amplifications of lfGHRHR and xGHRHR.**
(PPTX)

Table S2 **Accession numbers of amino acid sequences used in the phylogenetic analysis** [27], [28], [29].
(PPTX)
