# Supplementary material for: Structural and Functional Divergence of Growth Hormone-Releasing Hormone Receptors in Early Sarcopterygians: Lungfish and Xenopus
Source: PLoS One. 2013 Jan 4;8(1):e53482. doi: 10.1371/journal.pone.0053482 (PMC3537680; doi:10.1371/journal.pone.0053482)
Supplement: Figure S1 — X. laevis growth hormone-releasing hormone receptor 2 (xGHRHR2) nucleotide (GenBank accession no. JN378527) and deduced amino acid sequences. Nucleotides (lower line) and amino acids (upper line) were numbered from the initiation methionine. The full-length cDNA was 1835 bp with an open reading frame of 1269 bp encoding a 423-amino acid protein. The signal peptide (29 amino acids) was indicated in bold characters. Transmembrane domains were underlined with solid lines. These structural features were determined by software from the CBS Prediction Servers (http://www.cbs.dtu.dk/services/). (PPTX) [file pone.0053482.s001.pptx]

## Slide 1
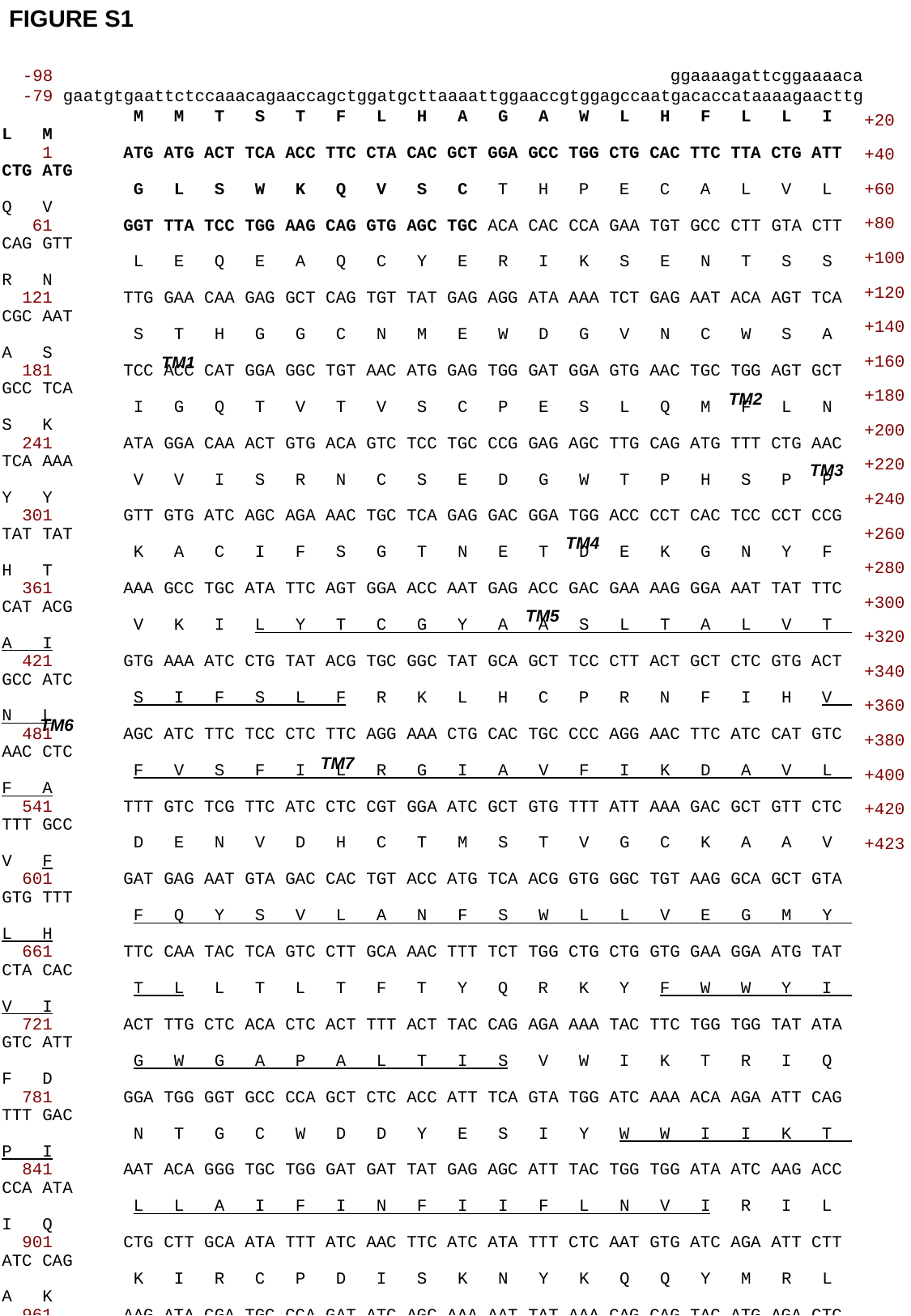

FIGURE S1
-98 ggaaaagattcggaaaaca
-79 gaatgtgaattctccaaacagaaccagctggatgcttaaaattggaaccgtggagccaatgacaccataaaagaacttg
+20
+40
+60
+80
+100
+120
+140
+160
+180
+200
+220
+240
+260
+280
+300
+320
+340
+360
+380
+400
+420
+423
	 M M T S T F L H A G A W L H F L L I L M
 1	ATG ATG ACT TCA ACC TTC CTA CAC GCT GGA GCC TGG CTG CAC TTC TTA CTG ATT CTG ATG
	 G L S W K Q V S C T H P E C A L V L Q V
 61	GGT TTA TCC TGG AAG CAG GTG AGC TGC ACA CAC CCA GAA TGT GCC CTT GTA CTT CAG GTT
	 L E Q E A Q C Y E R I K S E N T S S R N
 121	TTG GAA CAA GAG GCT CAG TGT TAT GAG AGG ATA AAA TCT GAG AAT ACA AGT TCA CGC AAT
	 S T H G G C N M E W D G V N C W S A A S
 181	TCC ACC CAT GGA GGC TGT AAC ATG GAG TGG GAT GGA GTG AAC TGC TGG AGT GCT GCC TCA
	 I G Q T V T V S C P E S L Q M F L N S K
 241	ATA GGA CAA ACT GTG ACA GTC TCC TGC CCG GAG AGC TTG CAG ATG TTT CTG AAC TCA AAA
	 V V I S R N C S E D G W T P H S P P Y Y
 301	GTT GTG ATC AGC AGA AAC TGC TCA GAG GAC GGA TGG ACC CCT CAC TCC CCT CCG TAT TAT
	 K A C I F S G T N E T D E K G N Y F H T
 361	AAA GCC TGC ATA TTC AGT GGA ACC AAT GAG ACC GAC GAA AAG GGA AAT TAT TTC CAT ACG
	 V K I L Y T C G Y A A S L T A L V T A I
 421	GTG AAA ATC CTG TAT ACG TGC GGC TAT GCA GCT TCC CTT ACT GCT CTC GTG ACT GCC ATC
	 S I F S L F R K L H C P R N F I H V N L
 481	AGC ATC TTC TCC CTC TTC AGG AAA CTG CAC TGC CCC AGG AAC TTC ATC CAT GTC AAC CTC
	 F V S F I L R G I A V F I K D A V L F A
 541	TTT GTC TCG TTC ATC CTC CGT GGA ATC GCT GTG TTT ATT AAA GAC GCT GTT CTC TTT GCC
	 D E N V D H C T M S T V G C K A A V V F
 601	GAT GAG AAT GTA GAC CAC TGT ACC ATG TCA ACG GTG GGC TGT AAG GCA GCT GTA GTG TTT
	 F Q Y S V L A N F S W L L V E G M Y L H
 661	TTC CAA TAC TCA GTC CTT GCA AAC TTT TCT TGG CTG CTG GTG GAA GGA ATG TAT CTA CAC
	 T L L T L T F T Y Q R K Y F W W Y I V I
 721	ACT TTG CTC ACA CTC ACT TTT ACT TAC CAG AGA AAA TAC TTC TGG TGG TAT ATA GTC ATT
	 G W G A P A L T I S V W I K T R I Q F D
 781	GGA TGG GGT GCC CCA GCT CTC ACC ATT TCA GTA TGG ATC AAA ACA AGA ATT CAG TTT GAC
	 N T G C W D D Y E S I Y W W I I K T P I
 841	AAT ACA GGG TGC TGG GAT GAT TAT GAG AGC ATT TAC TGG TGG ATA ATC AAG ACC CCA ATA
	 L L A I F I N F I I F L N V I R I L I Q
 901	CTG CTT GCA ATA TTT ATC AAC TTC ATC ATA TTT CTC AAT GTG ATC AGA ATT CTT ATC CAG
	 K I R C P D I S K N Y K Q Q Y M R L A K
 961	AAG ATA CGA TGC CCA GAT ATC AGC AAA AAT TAT AAA CAG CAG TAC ATG AGA CTC GCA AAG
	 S T L L L I P L F G V H Y V I F A L F P
 1021	TCT ACT CTT CTC CTC ATC CCT CTG TTT GGG GTT CAT TAT GTT ATC TTC GCA CTG TTT CCG
	 E H I G I W A R M Y F E L V L G S N Q G
 1081	GAG CAC ATC GGT ATT TGG GCA CGA ATG TAC TTT GAA CTT GTT CTT GGA TCC AAT CAG GGA
	 F I V A L L Y C F L N G E V Q A E I Q R
 1141	TTC ATA GTG GCT TTG CTC TAC TGC TTC CTC AAT GGA GAG GTC CAG GCG GAG ATC CAG AGG
	 H W G K W Q S S L E S N V F N L V T Q D
 1201	CAC TGG GGC AAG TGG CAG AGC TCA TTA GAG AGC AAC GTA TTT AAC CTG GTG ACA CAA GAC
	 F T A *
 1261	TTC ACA GCA TAAagtcataggcactgaagatgcaaagaagaccttttaggatggtacaaagcgcaaagaagacctt
 1337	ttagggtgctacaaagcagggctaaccgtaaacccggcaaaaattaaatgcagagcttgctatgntatttatcaagcaa
 1416 caacccatcaactcttcattgggccccctcttatcatcagctatgacacaagttgcattgtgctcggtttctaatgaca
 1495 tcatgagtctatccatagtgagtggaatatagaagtccaattggggggtttgcagtctccaaaagctggtaatgatagc
 1574	aatgtacctagacccttttgaggaatctgtctgcatcatactgatgcgngttttcatcataccccttcatctgctataa
 1653	gtacccccataatctctttgtcctaacaatttcaccccatattaaggggcatattgtatttaaactctgaacttaaaaa
 1732 aaaaaaaaaaaaaaaaa
TM1
TM2
TM3
TM4
TM5
TM6
TM7
