# Supplementary material for: Structural and Functional Divergence of Growth Hormone-Releasing Hormone Receptors in Early Sarcopterygians: Lungfish and Xenopus
Source: PLoS One. 2013 Jan 4;8(1):e53482. doi: 10.1371/journal.pone.0053482 (PMC3537680; doi:10.1371/journal.pone.0053482)
Supplement: Figure S2 — Lungfish P. dolloi growth hormone-releasing hormone receptor (lfGHRHR) nucleotide (GenBank accession no. JN378526) and deduced amino acid sequences. Nucleotides (lower line) and amino acids (upper line) were numbered from the initiation methionine. The full-length cDNA was 2191 bp with an open reading frame of 1308 bp encoding a 436-amino acid protein. The signal peptide (29 amino acids) was indicated in bold characters. Transmembrane domains were underlined with solid lines. (PPTX) [file pone.0053482.s002.pptx]

## Slide 1
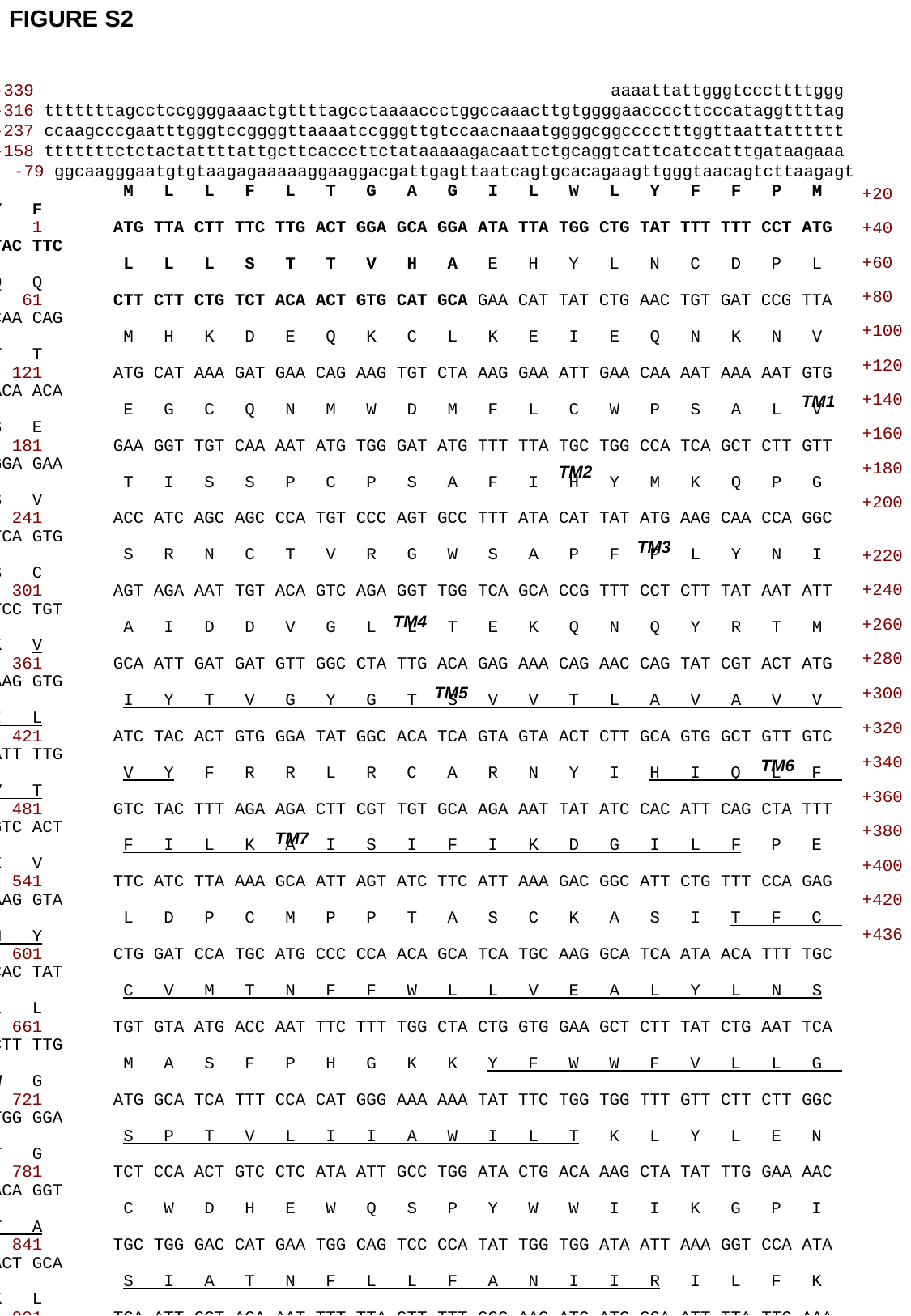

FIGURE S2
-339 aaaattattgggtcccttttggg
-316 tttttttagcctccggggaaactgttttagcctaaaaccctggccaaacttgtggggaaccccttcccataggttttag
-237 ccaagcccgaatttgggtccggggttaaaatccgggttgtccaacnaaatggggcggcccctttggttaattatttttt
-158 tttttttctctactattttattgcttcacccttctataaaaagacaattctgcaggtcattcatccatttgataagaaa
-79 ggcaagggaatgtgtaagagaaaaaggaaggacgattgagttaatcagtgcacagaagttgggtaacagtcttaagagt
+20
+40
+60
+80
+100
+120
+140
+160
+180
+200
	 M L L F L T G A G I L W L Y F F P M Y F
 1	ATG TTA CTT TTC TTG ACT GGA GCA GGA ATA TTA TGG CTG TAT TTT TTT CCT ATG TAC TTC
	 L L L S T T V H A E H Y L N C D P L Q Q
 61	CTT CTT CTG TCT ACA ACT GTG CAT GCA GAA CAT TAT CTG AAC TGT GAT CCG TTA CAA CAG
	 M H K D E Q K C L K E I E Q N K N V T T
 121	ATG CAT AAA GAT GAA CAG AAG TGT CTA AAG GAA ATT GAA CAA AAT AAA AAT GTG ACA ACA
	 E G C Q N M W D M F L C W P S A L V G E
 181	GAA GGT TGT CAA AAT ATG TGG GAT ATG TTT TTA TGC TGG CCA TCA GCT CTT GTT GGA GAA
	 T I S S P C P S A F I H Y M K Q P G S V
 241	ACC ATC AGC AGC CCA TGT CCC AGT GCC TTT ATA CAT TAT ATG AAG CAA CCA GGC TCA GTG
	 S R N C T V R G W S A P F P L Y N I S C
 301	AGT AGA AAT TGT ACA GTC AGA GGT TGG TCA GCA CCG TTT CCT CTT TAT AAT ATT TCC TGT
	 A I D D V G L L T E K Q N Q Y R T M K V
 361	GCA ATT GAT GAT GTT GGC CTA TTG ACA GAG AAA CAG AAC CAG TAT CGT ACT ATG AAG GTG
	 I Y T V G Y G T S V V T L A V A V V I L
 421	ATC TAC ACT GTG GGA TAT GGC ACA TCA GTA GTA ACT CTT GCA GTG GCT GTT GTC ATT TTG
	 V Y F R R L R C A R N Y I H I Q L F V T
 481	GTC TAC TTT AGA AGA CTT CGT TGT GCA AGA AAT TAT ATC CAC ATT CAG CTA TTT GTC ACT
	 F I L K A I S I F I K D G I L F P E K V
 541	TTC ATC TTA AAA GCA ATT AGT ATC TTC ATT AAA GAC GGC ATT CTG TTT CCA GAG AAG GTA
	 L D P C M P P T A S C K A S I T F C H Y
 601	CTG GAT CCA TGC ATG CCC CCA ACA GCA TCA TGC AAG GCA TCA ATA ACA TTT TGC CAC TAT
	 C V M T N F F W L L V E A L Y L N S L L
 661	TGT GTA ATG ACC AAT TTC TTT TGG CTA CTG GTG GAA GCT CTT TAT CTG AAT TCA CTT TTG
	 M A S F P H G K K Y F W W F V L L G W G
 721	ATG GCA TCA TTT CCA CAT GGG AAA AAA TAT TTC TGG TGG TTT GTT CTT CTT GGC TGG GGA
	 S P T V L I I A W I L T K L Y L E N T G
 781	TCT CCA ACT GTC CTC ATA ATT GCC TGG ATA CTG ACA AAG CTA TAT TTG GAA AAC ACA GGT
	 C W D H E W Q S P Y W W I I K G P I T A
 841	TGC TGG GAC CAT GAA TGG CAG TCC CCA TAT TGG TGG ATA ATT AAA GGT CCA ATA ACT GCA
	 S I A T N F L L F A N I I R I L F K K L
 901	TCA ATT GCT ACA AAT TTT TTA CTT TTT GCC AAC ATC ATC CGA ATT TTA TTC AAA AAG CTC
	 D P R K I N F N N S F Q Y R R L S K S T
 961	GAC CCT AGA AAG ATC AAC TTT AAT AAC TCA TTT CAA TAC AGG CGT TTA TCA AAA TCA ACT
	 L L L I P L F G T H Y I V F N F L P E Y
 1021	CTA CTT CTA ATC CCA CTG TTT GGA ACG CAC TAC ATT GTT TTT AAC TTC CTT CCA GAG TAC
	 I H V E G R L Y L E L C L G S F Q G F M
 1081	ATC CAC GTG GAA GGT AGA CTG TAT TTG GAA CTC TGT CTT GGT TCA TTT CAG GGC TTC ATG
	 V A V L Y C F L N Q E V Q A E I T R K W
 1141	GTC GCT GTT CTT TAC TGC TTC CTG AAT CAA GAG GTG CAG GCA GAA ATT ACT CGG AAG TGG
	 Q R M H I N S Y G L V P F T A K D S Q R
 1201	CAA CGA ATG CAT ATC AAT AGC TAT GGG CTT GTA CCG TTT ACT GCT AAA GAC AGT CAA AGG
	 T L R S S L G G M K I S T S E C *
 1261	ACT TTA CGA TCC AGC TTA GGA GGA ATG AAA ATT AGC ACA TCT GAA TGC TAGataacagacacc
 1324	tttgtcatttaaaaactcttccctgcctatgctttgtgttgactgaaatgttcccattcagaggaaggaagccacttca
 1403 actataatgtctgcagtttaaagataggatcgttcagtttcaagatggagaaatgctatcaacttgttaaggccaagcc
 1482	tttaggaatgctaagacatttagtgacatgaaaattagtttcttacttggagtcagaatgctttacagactcaatagtt
 1561	ctgaaacgtaagacccatgttgtaacaattgtagtagtagttaaattgctccactatttactacactgcagttttgtta
 1640	gactttttggttattaaacaatgtgcaattacctgtgaaaattcagtgcacattagtcatgctgtaatatattattaca
 1719	aaatattgtgctgtaaaaagaaactatttgttttagcgtagcatgttatggtacactgctaaagtttagctttttgtag
 1798	tgattgttctaattacaaaacaaatctctaaaatgtgaaaaaaaaaaaaaaaaa
+220
+240
+260
+280
+300
+320
+340
+360
+380
+400
+420
+436
TM1
TM2
TM3
TM4
TM5
TM6
TM7
