# Supplementary figures and images for: Structural and Functional Divergence of Growth Hormone-Releasing Hormone Receptors in Early Sarcopterygians: Lungfish and Xenopus
Source: PLoS One. 2013 Jan 4;8(1):e53482. doi: 10.1371/journal.pone.0053482 (PMC3537680; doi:10.1371/journal.pone.0053482)

## Slide 1
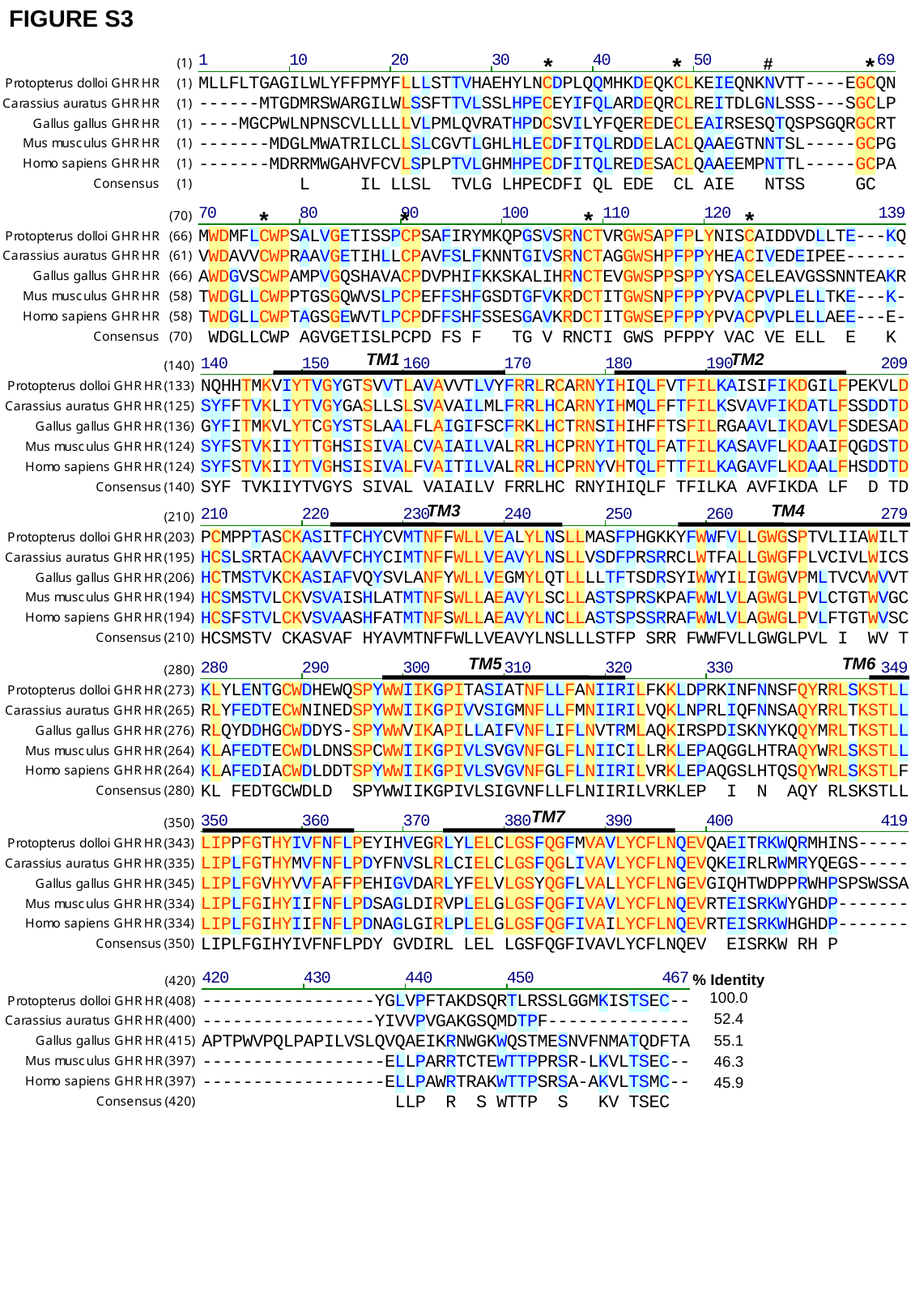

FIGURE S3
*
*
*
#
*
*
*
*
TM1
TM2
TM3
TM4
TM5
TM6
TM7
| % Identity |
| --- |
| 100.0 |
| 52.4 |
| 55.1 |
| 46.3 |
| 45.9 |

Supplement: Figure S3 — Growth hormone-releasing hormone receptor (GHRHR) protein sequence alignment with lfGHRHR. Identical and conserved amino acid residues were written and highlighted in orange and blue respectively. Putative transmembrane domains were overlined and labeled. # and * indicate potential sites for N-linked glycosylation and conserved cysteine residues, respectively. Gaps (represented by - ) were introduced to maximize sequence homology. Percent amino acid identity and homology were listed in respect to lungfish GHRHR. (PPTX) [file pone.0053482.s003.pptx]

## Slide 1
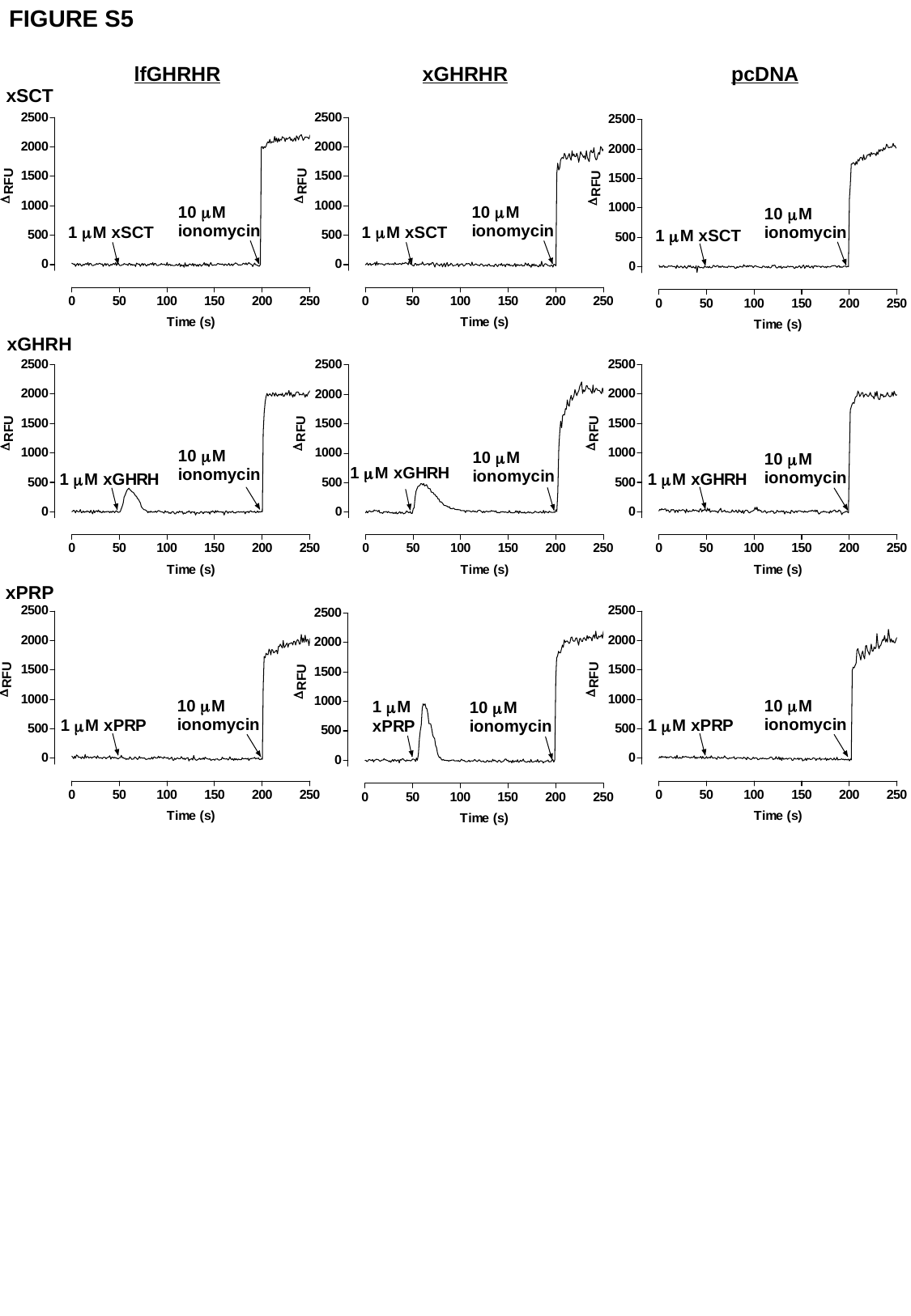

FIGURE S5
lfGHRHR
xGHRHR
pcDNA
xSCT
xGHRH
xPRP

Supplement: Figure S5 — Representative traces of xSCT, xGHRH and xPRP on intracellular calcium mobilization in lfGHRHR, xGHRHR and null pcDNA 3.1-transfected CHO cells. Peak magnitude of traces was proportional to the order of potency of the ligands tested. Traces were obtained from at least 10 calcium assays with respective control shown on the right panel. Ionomycin (10 µM) was added at the end of each experiment to test the vitality of the cells. (PPTX) [file pone.0053482.s005.pptx]
