# Supplementary material for: Structural and Functional Divergence of Growth Hormone-Releasing Hormone Receptors in Early Sarcopterygians: Lungfish and Xenopus
Source: PLoS One. 2013 Jan 4;8(1):e53482. doi: 10.1371/journal.pone.0053482 (PMC3537680; doi:10.1371/journal.pone.0053482)
Supplement: Figure S4 — Amino acid sequence comparison of X. laevis growth hormone-releasing hormone receptor (xGHRHR) to zebrafish D. rerio and chicken G. gallus GHRHR2 and PRPR. Identical and conserved amino acid residues were written and highlighted in orange and blue respectively. Putative transmembrane domains were overlined and labeled. # and * indicate potential sites for N-linked glycosylation and conserved cysteine residues, respectively. Gaps (represented by - ) were introduced to maximize sequence homology. Percent amino acid identity and homology were listed in respect to X. laevis GHRHR. (PPTX) [file pone.0053482.s004.pptx]

## Slide 1
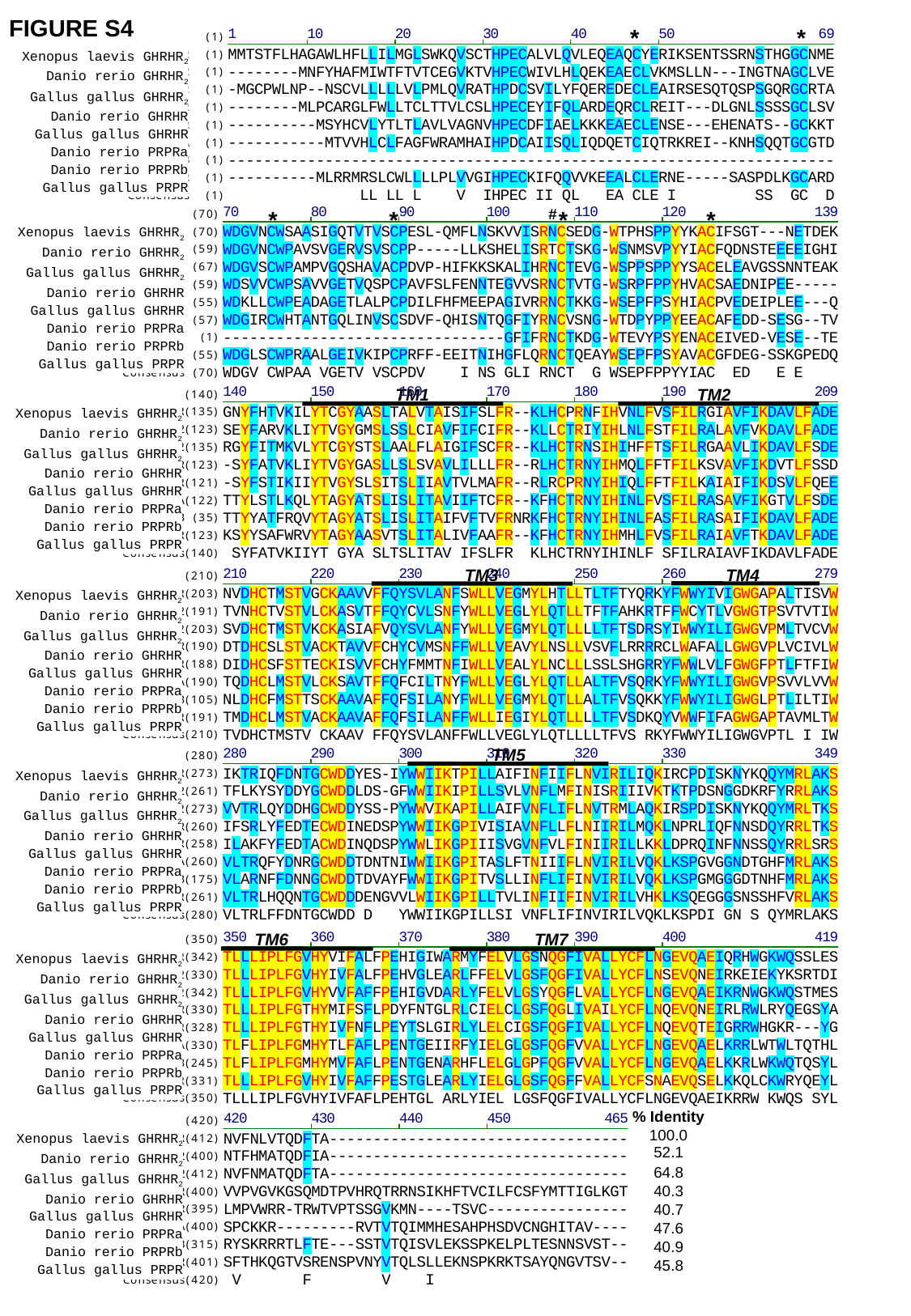

FIGURE S4
*
*
Xenopus laevis GHRHR2
Danio rerio GHRHR2
Gallus gallus GHRHR2
Danio rerio GHRHR
Gallus gallus GHRHR
Danio rerio PRPRa
Danio rerio PRPRb
Gallus gallus PRPR
*
*
*
#
*
Xenopus laevis GHRHR2
Danio rerio GHRHR2
Gallus gallus GHRHR2
Danio rerio GHRHR
Gallus gallus GHRHR
Danio rerio PRPRa
Danio rerio PRPRb
Gallus gallus PRPR
TM1
TM2
Xenopus laevis GHRHR2
Danio rerio GHRHR2
Gallus gallus GHRHR2
Danio rerio GHRHR
Gallus gallus GHRHR
Danio rerio PRPRa
Danio rerio PRPRb
Gallus gallus PRPR
TM3
TM4
Xenopus laevis GHRHR2
Danio rerio GHRHR2
Gallus gallus GHRHR2
Danio rerio GHRHR
Gallus gallus GHRHR
Danio rerio PRPRa
Danio rerio PRPRb
Gallus gallus PRPR
TM5
Xenopus laevis GHRHR2
Danio rerio GHRHR2
Gallus gallus GHRHR2
Danio rerio GHRHR
Gallus gallus GHRHR
Danio rerio PRPRa
Danio rerio PRPRb
Gallus gallus PRPR
TM5
TM6
TM7
Xenopus laevis GHRHR2
Danio rerio GHRHR2
Gallus gallus GHRHR2
Danio rerio GHRHR
Gallus gallus GHRHR
Danio rerio PRPRa
Danio rerio PRPRb
Gallus gallus PRPR
Xenopus laevis GHRHR2
Danio rerio GHRHR2
Gallus gallus GHRHR2
Danio rerio GHRHR
Gallus gallus GHRHR
Danio rerio PRPRa
Danio rerio PRPRb
Gallus gallus PRPR
| % Identity |
| --- |
| 100.0 52.1 |
| 64.8 |
| 40.3 |
| 40.7 |
| 47.6 |
| 40.9 |
| 45.8 |
