# Supplementary material for: Structural and Functional Divergence of Growth Hormone-Releasing Hormone Receptors in Early Sarcopterygians: Lungfish and Xenopus
Source: PLoS One. 2013 Jan 4;8(1):e53482. doi: 10.1371/journal.pone.0053482 (PMC3537680; doi:10.1371/journal.pone.0053482)
Supplement: Figure S6 — Chromosomal synteny analysis of GHRHR and PRPR in osteichthyans. Genes in vicinity of GHRHR and/or PRPR were mapped, and syntenic genes were linked by straight lines. Size of the chromosomal region analyzed was given underneath based on the current edition of Ensembl databases. Syntenic genes encoding secretin GPCR receptors were drawn in bold, NEUROD6 and XCR1 were written in colour fonts for easy referencing to main text. (PPTX) [file pone.0053482.s006.pptx]

## Slide 1
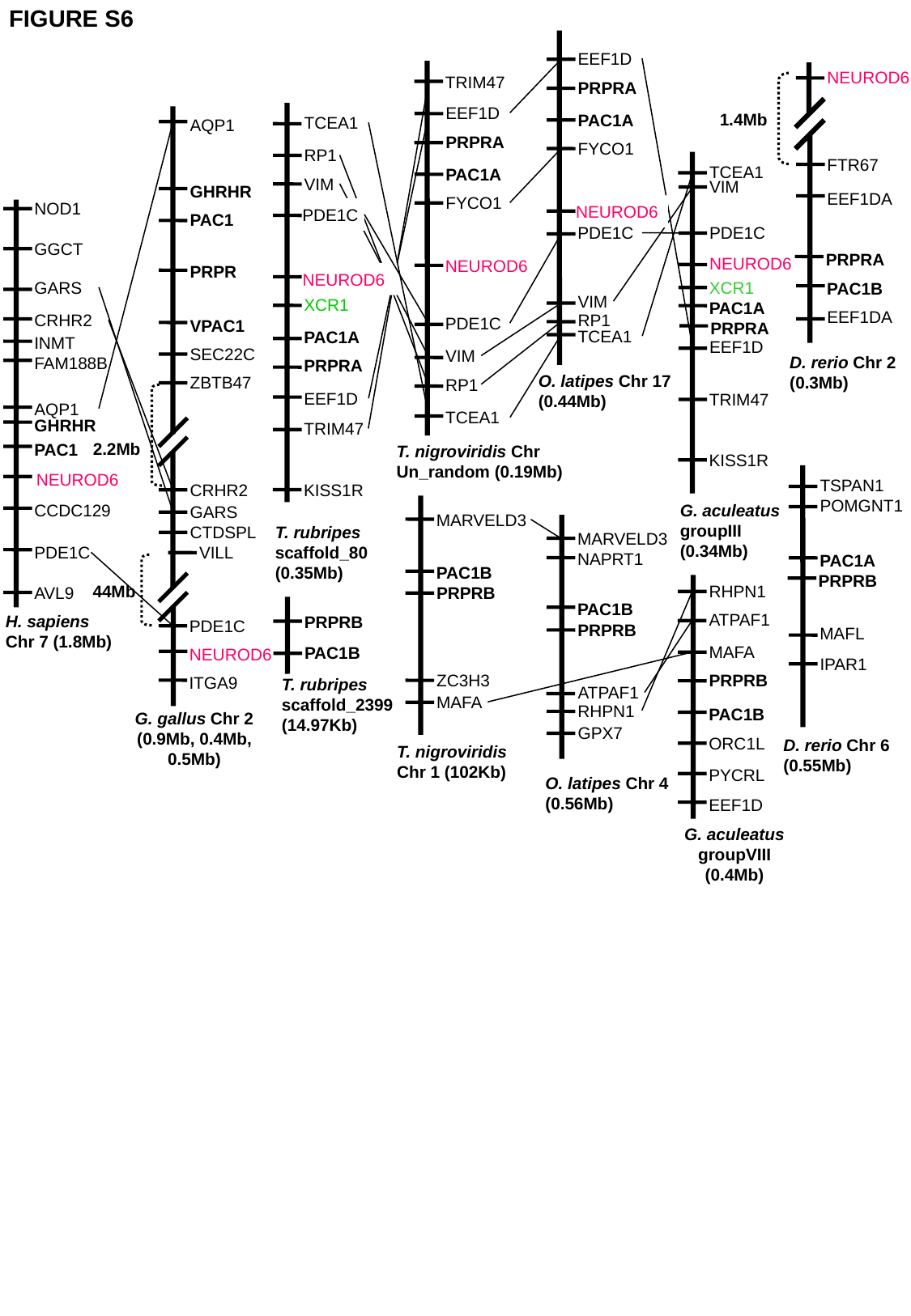

FIGURE S6
EEF1D
PRPRA
PAC1A
FYCO1
NEUROD6
PDE1C
VIM
RP1
TCEA1
TRIM47
EEF1D
PRPRA
PAC1A
FYCO1
NEUROD6
PDE1C
VIM
RP1
TCEA1
NEUROD6
1.4Mb
FTR67
EEF1DA
PRPRA
PAC1B
EEF1DA
D. rerio Chr 2 (0.3Mb)
TCEA1
RP1
VIM
PDE1C
NEUROD6
XCR1
PAC1A
PRPRA
EEF1D
TRIM47
KISS1R
AQP1
GHRHR
PAC1
PRPR
VPAC1
SEC22C
ZBTB47
CRHR2
GARS
CTDSPL
PDE1C
NEUROD6
ITGA9
2.2Mb
44Mb
TCEA1
VIM
PDE1C
NEUROD6
XCR1
PAC1A
PRPRA
EEF1D
TRIM47
KISS1R
NOD1
GGCT
GARS
CRHR2
INMT
FAM188B
AQP1
GHRHR
PAC1
NEUROD6
CCDC129
PDE1C
AVL9
O. latipes Chr 17 (0.44Mb)
T. nigroviridis Chr Un_random (0.19Mb)
TSPAN1
POMGNT1
PAC1A
PRPRB
MAFL
IPAR1
D. rerio Chr 6 (0.55Mb)
G. aculeatus groupIII (0.34Mb)
MARVELD3
PAC1B
PRPRB
ZC3H3
MAFA
MARVELD3
NAPRT1
PAC1B
PRPRB
ATPAF1
RHPN1
GPX7
T. rubripes scaffold_80 (0.35Mb)
VILL
RHPN1
ATPAF1
MAFA
PRPRB
PAC1B
ORC1L
PYCRL
EEF1D
PRPRB
PAC1B
H. sapiens Chr 7 (1.8Mb)
T. rubripes scaffold_2399 (14.97Kb)
G. gallus Chr 2 (0.9Mb, 0.4Mb, 0.5Mb)
T. nigroviridis Chr 1 (102Kb)
O. latipes Chr 4 (0.56Mb)
G. aculeatus groupVIII (0.4Mb)
