# Supplementary material for: Structural and Functional Divergence of Growth Hormone-Releasing Hormone Receptors in Early Sarcopterygians: Lungfish and Xenopus
Source: PLoS One. 2013 Jan 4;8(1):e53482. doi: 10.1371/journal.pone.0053482 (PMC3537680; doi:10.1371/journal.pone.0053482)
Supplement: Figure S7 — Amino acid sequence comparison of GHRH. The coelacanth GHRH was predicted from the newly released genome sequence. (PPTX) [file pone.0053482.s007.pptx]

## Slide 1
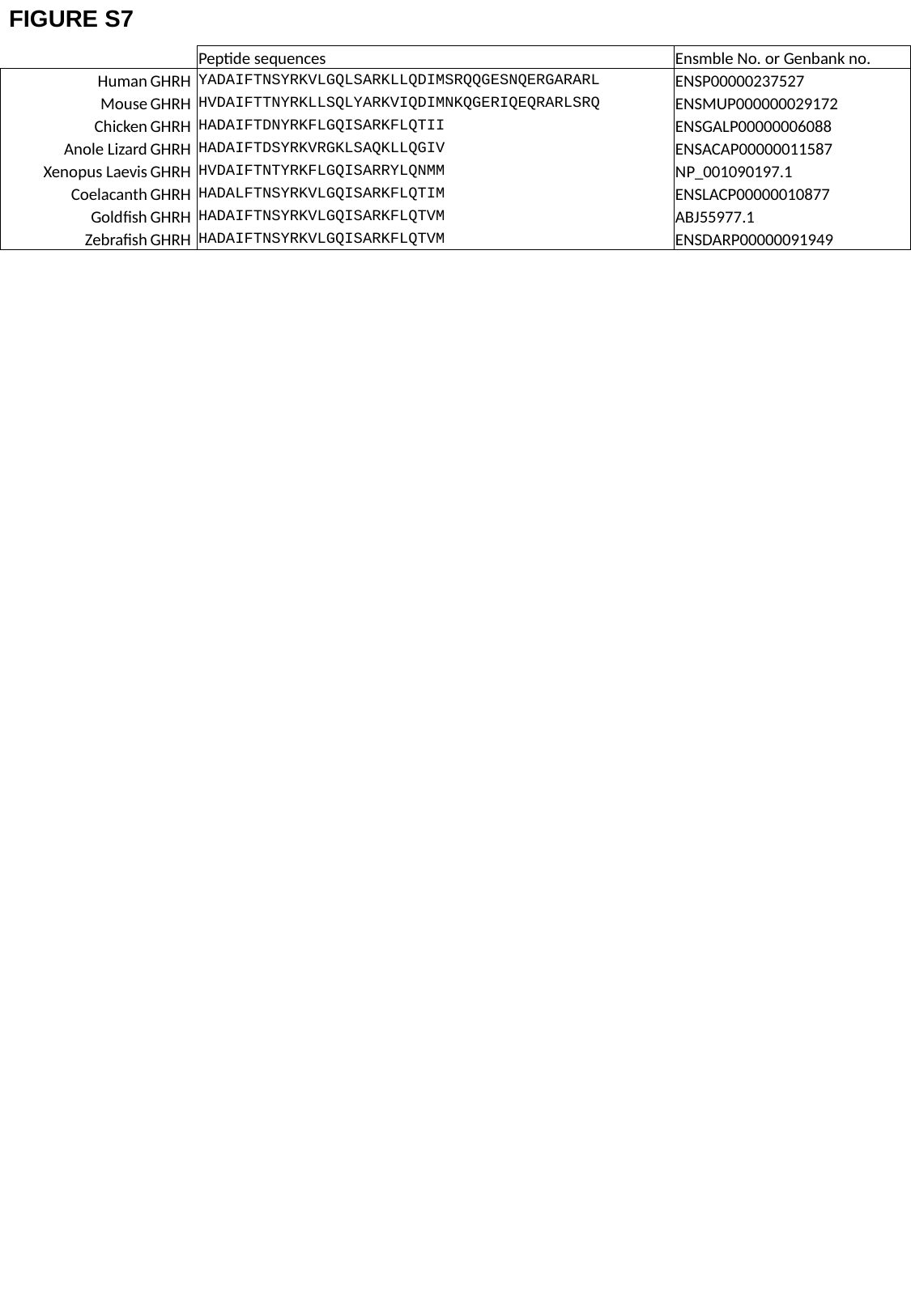

FIGURE S7
| | | Peptide sequences | Ensmble No. or Genbank no. |
| --- | --- | --- | --- |
| Human | GHRH | YADAIFTNSYRKVLGQLSARKLLQDIMSRQQGESNQERGARARL | ENSP00000237527 |
| Mouse | GHRH | HVDAIFTTNYRKLLSQLYARKVIQDIMNKQGERIQEQRARLSRQ | ENSMUP000000029172 |
| Chicken | GHRH | HADAIFTDNYRKFLGQISARKFLQTII | ENSGALP00000006088 |
| Anole Lizard | GHRH | HADAIFTDSYRKVRGKLSAQKLLQGIV | ENSACAP00000011587 |
| Xenopus Laevis | GHRH | HVDAIFTNTYRKFLGQISARRYLQNMM | NP\_001090197.1 |
| Coelacanth | GHRH | HADALFTNSYRKVLGQISARKFLQTIM | ENSLACP00000010877 |
| Goldfish | GHRH | HADAIFTNSYRKVLGQISARKFLQTVM | ABJ55977.1 |
| Zebrafish | GHRH | HADAIFTNSYRKVLGQISARKFLQTVM | ENSDARP00000091949 |
