# Supplementary material for: Structural and Functional Divergence of Growth Hormone-Releasing Hormone Receptors in Early Sarcopterygians: Lungfish and Xenopus
Source: PLoS One. 2013 Jan 4;8(1):e53482. doi: 10.1371/journal.pone.0053482 (PMC3537680; doi:10.1371/journal.pone.0053482)
Supplement: Table S1 — List of primers used in PCR and real-time PCR amplifications of lfGHRHR and xGHRHR. (PPTX) [file pone.0053482.s008.pptx]

## Slide 1
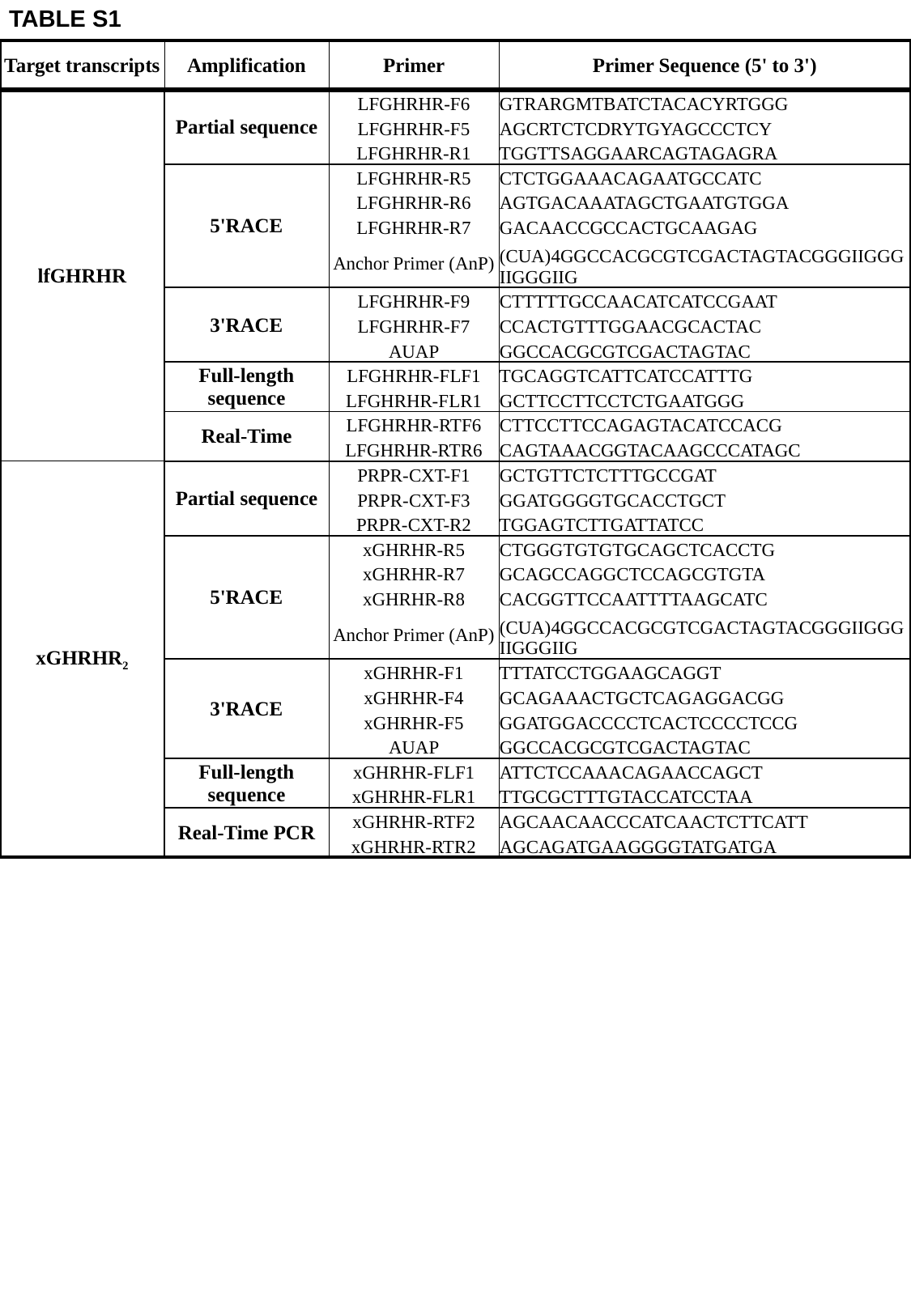

TABLE S1
| Target transcripts | Amplification | Primer | Primer Sequence (5' to 3') |
| --- | --- | --- | --- |
| lfGHRHR | Partial sequence | LFGHRHR-F6 | GTRARGMTBATCTACACYRTGGG |
| | | LFGHRHR-F5 | AGCRTCTCDRYTGYAGCCCTCY |
| | | LFGHRHR-R1 | TGGTTSAGGAARCAGTAGAGRA |
| | 5'RACE | LFGHRHR-R5 | CTCTGGAAACAGAATGCCATC |
| | | LFGHRHR-R6 | AGTGACAAATAGCTGAATGTGGA |
| | | LFGHRHR-R7 | GACAACCGCCACTGCAAGAG |
| | | Anchor Primer (AnP) | (CUA)4GGCCACGCGTCGACTAGTACGGGIIGGGIIGGGIIG |
| | 3'RACE | LFGHRHR-F9 | CTTTTTGCCAACATCATCCGAAT |
| | | LFGHRHR-F7 | CCACTGTTTGGAACGCACTAC |
| | | AUAP | GGCCACGCGTCGACTAGTAC |
| | Full-length sequence | LFGHRHR-FLF1 | TGCAGGTCATTCATCCATTTG |
| | | LFGHRHR-FLR1 | GCTTCCTTCCTCTGAATGGG |
| | Real-Time | LFGHRHR-RTF6 | CTTCCTTCCAGAGTACATCCACG |
| | | LFGHRHR-RTR6 | CAGTAAACGGTACAAGCCCATAGC |
| xGHRHR2 | Partial sequence | PRPR-CXT-F1 | GCTGTTCTCTTTGCCGAT |
| | | PRPR-CXT-F3 | GGATGGGGTGCACCTGCT |
| | | PRPR-CXT-R2 | TGGAGTCTTGATTATCC |
| | 5'RACE | xGHRHR-R5 | CTGGGTGTGTGCAGCTCACCTG |
| | | xGHRHR-R7 | GCAGCCAGGCTCCAGCGTGTA |
| | | xGHRHR-R8 | CACGGTTCCAATTTTAAGCATC |
| | | Anchor Primer (AnP) | (CUA)4GGCCACGCGTCGACTAGTACGGGIIGGGIIGGGIIG |
| | 3'RACE | xGHRHR-F1 | TTTATCCTGGAAGCAGGT |
| | | xGHRHR-F4 | GCAGAAACTGCTCAGAGGACGG |
| | | xGHRHR-F5 | GGATGGACCCCTCACTCCCCTCCG |
| | | AUAP | GGCCACGCGTCGACTAGTAC |
| | Full-length sequence | xGHRHR-FLF1 | ATTCTCCAAACAGAACCAGCT |
| | | xGHRHR-FLR1 | TTGCGCTTTGTACCATCCTAA |
| | Real-Time PCR | xGHRHR-RTF2 | AGCAACAACCCATCAACTCTTCATT |
| | | xGHRHR-RTR2 | AGCAGATGAAGGGGTATGATGA |
