# Supplementary material for: Structural and Functional Divergence of Growth Hormone-Releasing Hormone Receptors in Early Sarcopterygians: Lungfish and Xenopus
Source: PLoS One. 2013 Jan 4;8(1):e53482. doi: 10.1371/journal.pone.0053482 (PMC3537680; doi:10.1371/journal.pone.0053482)
Supplement: Table S2 — Accession numbers of amino acid sequences used in the phylogenetic analysis [27], [28], [29]. (PPTX) [file pone.0053482.s009.pptx]

## Slide 1
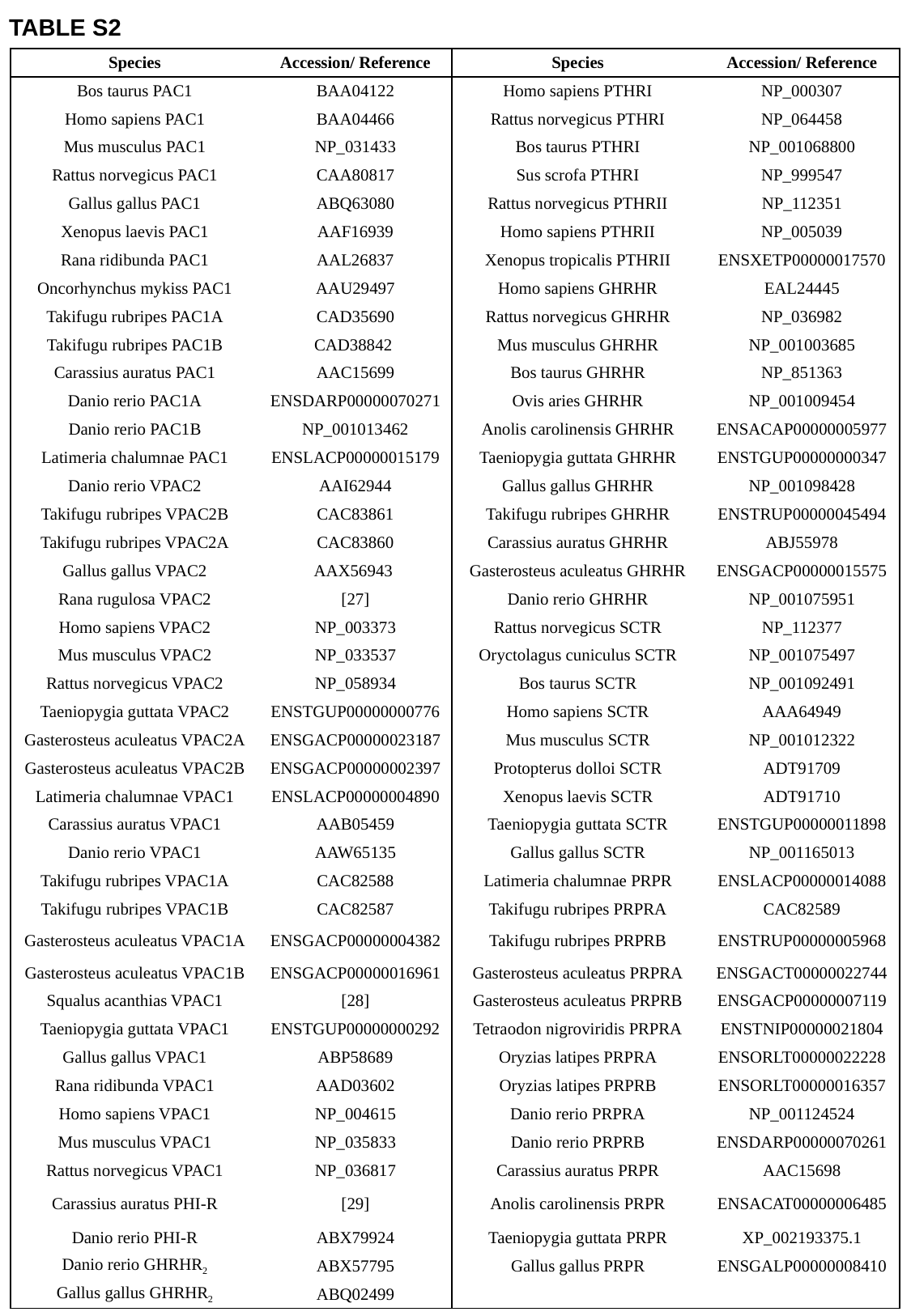

TABLE S2
| Species | Accession/ Reference | Species | Accession/ Reference |
| --- | --- | --- | --- |
| Bos taurus PAC1 | BAA04122 | Homo sapiens PTHRI | NP\_000307 |
| Homo sapiens PAC1 | BAA04466 | Rattus norvegicus PTHRI | NP\_064458 |
| Mus musculus PAC1 | NP\_031433 | Bos taurus PTHRI | NP\_001068800 |
| Rattus norvegicus PAC1 | CAA80817 | Sus scrofa PTHRI | NP\_999547 |
| Gallus gallus PAC1 | ABQ63080 | Rattus norvegicus PTHRII | NP\_112351 |
| Xenopus laevis PAC1 | AAF16939 | Homo sapiens PTHRII | NP\_005039 |
| Rana ridibunda PAC1 | AAL26837 | Xenopus tropicalis PTHRII | ENSXETP00000017570 |
| Oncorhynchus mykiss PAC1 | AAU29497 | Homo sapiens GHRHR | EAL24445 |
| Takifugu rubripes PAC1A | CAD35690 | Rattus norvegicus GHRHR | NP\_036982 |
| Takifugu rubripes PAC1B | CAD38842 | Mus musculus GHRHR | NP\_001003685 |
| Carassius auratus PAC1 | AAC15699 | Bos taurus GHRHR | NP\_851363 |
| Danio rerio PAC1A | ENSDARP00000070271 | Ovis aries GHRHR | NP\_001009454 |
| Danio rerio PAC1B | NP\_001013462 | Anolis carolinensis GHRHR | ENSACAP00000005977 |
| Latimeria chalumnae PAC1 | ENSLACP00000015179 | Taeniopygia guttata GHRHR | ENSTGUP00000000347 |
| Danio rerio VPAC2 | AAI62944 | Gallus gallus GHRHR | NP\_001098428 |
| Takifugu rubripes VPAC2B | CAC83861 | Takifugu rubripes GHRHR | ENSTRUP00000045494 |
| Takifugu rubripes VPAC2A | CAC83860 | Carassius auratus GHRHR | ABJ55978 |
| Gallus gallus VPAC2 | AAX56943 | Gasterosteus aculeatus GHRHR | ENSGACP00000015575 |
| Rana rugulosa VPAC2 | [27] | Danio rerio GHRHR | NP\_001075951 |
| Homo sapiens VPAC2 | NP\_003373 | Rattus norvegicus SCTR | NP\_112377 |
| Mus musculus VPAC2 | NP\_033537 | Oryctolagus cuniculus SCTR | NP\_001075497 |
| Rattus norvegicus VPAC2 | NP\_058934 | Bos taurus SCTR | NP\_001092491 |
| Taeniopygia guttata VPAC2 | ENSTGUP00000000776 | Homo sapiens SCTR | AAA64949 |
| Gasterosteus aculeatus VPAC2A | ENSGACP00000023187 | Mus musculus SCTR | NP\_001012322 |
| Gasterosteus aculeatus VPAC2B | ENSGACP00000002397 | Protopterus dolloi SCTR | ADT91709 |
| Latimeria chalumnae VPAC1 | ENSLACP00000004890 | Xenopus laevis SCTR | ADT91710 |
| Carassius auratus VPAC1 | AAB05459 | Taeniopygia guttata SCTR | ENSTGUP00000011898 |
| Danio rerio VPAC1 | AAW65135 | Gallus gallus SCTR | NP\_001165013 |
| Takifugu rubripes VPAC1A | CAC82588 | Latimeria chalumnae PRPR | ENSLACP00000014088 |
| Takifugu rubripes VPAC1B | CAC82587 | Takifugu rubripes PRPRA | CAC82589 |
| Gasterosteus aculeatus VPAC1A | ENSGACP00000004382 | Takifugu rubripes PRPRB | ENSTRUP00000005968 |
| Gasterosteus aculeatus VPAC1B | ENSGACP00000016961 | Gasterosteus aculeatus PRPRA | ENSGACT00000022744 |
| Squalus acanthias VPAC1 | [28] | Gasterosteus aculeatus PRPRB | ENSGACP00000007119 |
| Taeniopygia guttata VPAC1 | ENSTGUP00000000292 | Tetraodon nigroviridis PRPRA | ENSTNIP00000021804 |
| Gallus gallus VPAC1 | ABP58689 | Oryzias latipes PRPRA | ENSORLT00000022228 |
| Rana ridibunda VPAC1 | AAD03602 | Oryzias latipes PRPRB | ENSORLT00000016357 |
| Homo sapiens VPAC1 | NP\_004615 | Danio rerio PRPRA | NP\_001124524 |
| Mus musculus VPAC1 | NP\_035833 | Danio rerio PRPRB | ENSDARP00000070261 |
| Rattus norvegicus VPAC1 | NP\_036817 | Carassius auratus PRPR | AAC15698 |
| Carassius auratus PHI-R | [29] | Anolis carolinensis PRPR | ENSACAT00000006485 |
| Danio rerio PHI-R | ABX79924 | Taeniopygia guttata PRPR | XP\_002193375.1 |
| Danio rerio GHRHR2 | ABX57795 | Gallus gallus PRPR | ENSGALP00000008410 |
| Gallus gallus GHRHR2 | ABQ02499 | | |
